# Shift in Tissue-Specific Immune Niches and CD137 Expression in Tuberculoma of Pembrolizumab-Treated Nasopharyngeal Carcinoma Patients

**DOI:** 10.3390/cancers16020268

**Published:** 2024-01-08

**Authors:** Ngar Woon Kam, Anthony Wing Ip Lo, Desmond Tae Yang Hung, Ho Ko, Ka Chun Wu, Dora Lai Wan Kwong, Ka On Lam, To Wai Leung, Chi Ming Che, Victor Ho Fun Lee

**Affiliations:** 1 Department of Clinical Oncology, School of Clinical Medicine, LKS Faculty of Medicine, The University of Hong Kong, Hong Kong 999077, China; yvonnekam@hksccb.hk (N.W.K.); u3007510@connect.hku.hk (D.T.Y.H.); eymonwu@hku.hk (K.C.W.); dlwkwong@hku.hk (D.L.W.K.); lamkaon@hku.hk (K.O.L.); leungtw@ha.org.hk (T.W.L.); 2 Laboratory of Synthetic Chemistry and Chemical Biology Limited, Hong Kong 999077, China; cmche@hku.hk; 3 Department of Pathology, Queen Mary Hospital, Hong Kong 999077, China; awilo.qmh@gmail.com; 4 Department of Medicine and Therapeutics, The Chinese University of Hong Kong, Shatin, Hong Kong 999077, China; ho.ko@alumni.ucl.ac.uk; 5 Li Ka Shing Institute of Health Sciences, Faculty of Medicine, The Chinese University of Hong Kong, Shatin, Hong Kong 999077, China; 6 Clinical Oncology Center, The University of Hong Kong-Shenzhen Hospital, Shenzhen 518000, China; 7 Department of Chemistry, Faculty of Science, The University of Hong Kong, Hong Kong 999077, China

**Keywords:** CD137, tissue-specific immune microenvironment, immune checkpoint inhibitors, macrophages, nasopharyngeal carcinoma, spatial, tuberculosis reactivation, CD8+ T cells, immune-related adverse effects

## Abstract

**Simple Summary:**

Despite that a paradoxical response of immune checkpoint inhibition (ICI) is tuberculosis (TB) reactivation, our knowledge of cross-compartmental immune cellularity in human tissues remains limited. Existing studies mainly focused on treatment-associated malignant transformation or TB persistence. Unifying cellular heterogeneity across healthy and pathological tissues is crucial for developing treatments that overcome ICIs-related adverse events. In this study, we highlight the complexity of anti-PD1 functions, which go beyond enhancing immunity solely within the tumor microenvironment (TME) and can be influenced by shifts in CD137-rich immune niches. Each pathological lesion exhibited a unique tissue-specific immune microenvironment (TSIME) associated with ICI, emphasizing the need to tailor cancer therapy considering the characteristics of each TSIME for effective treatment. We also provide evidence of a niche-shift in the CD137-rich hostile TSIME, which may contribute to the reactivation of TB associated with ICI treatment. These findings serve as proof-of-concept for the potential clinical development of selectively activating anti-CD137 signaling within the TME, in combination with checkpoint blockade.

**Abstract:**

The use of immune checkpoint inhibitors (ICIs) in cancer treatment has shown promise but can also have unintended consequences, such as reactivating latent tuberculosis (TB). To develop treatments that address ICIs-related adverse events, it is essential to understand cellular heterogeneity across healthy and pathological tissues. We performed cross-tissue multiplexed staining analysis on samples from two patients with TB reactivation during pembrolizumab treatment for metastatic nasopharyngeal carcinoma. CD8+ T cells, rather than CD4+ T cells, accumulated preferentially in the tuberculoma and were associated with increased production of IFNγ and expression of CD137. Additionally, CD137 enrichment played a role in the spatial organization of the tuberculoma, with specific interaction limited to spatial proximal cells between IFNγ+ CD137+ CD8+ T cells and IL12+ CD137+ type-1 macrophages. This unique feature was not observed in non-tumoral or tumoral tissues. Our analysis of public transcriptomic datasets supported the notion that this cellular interaction was more prominent in patients with durable ICI responses compared to those with non-ICI-related TB. We suggest that shifts towards CD137-rich immune niches are correlated with both off-target immune-related adverse events and anti-tumor efficacy. Targeting the tumor microenvironment through conditional activation of anti-CD137 signaling in combination with ICIs can modulate the reactivity of T cells and macrophages for localized tumor killing without the potential off-target immune-related risks associated with ICIs alone.

## 1. Introduction

Locally advanced nasopharyngeal carcinoma (NPC) is typically treated with chemoradiation, but relapse occurs in about 30% of patients [1]. Immune checkpoint inhibitors (ICIs) like nivolumab and pembrolizumab have revolutionized cancer treatment by targeting PD-1 to restore antitumor effects in the tumor microenvironment (TME) [2]. However, the use of ICIs has been associated with immune-related adverse events (irAEs) [3], including the rare but clinically significant event of tuberculosis reactivation (TB) [4,5]. TB is caused by Mycobacterium tuberculosis (MTB) infection, which can remain dormant for long periods. While T-cell responses are critical for TB immunity in humans, binding of PD-1 to its ligands provides a negative feedback signal to counteract T cell receptor activation. Although PD-1 inhibition was expected to enhance immune activation and control both cancer and bacterial infection, clinical experiences have shown that TB reactivation occurs in cancer patients who respond well to ICIs. The underlying process of TB reactivation in these patients is not yet fully understood [6,7,8,9,10,11,12]. 

Previous studies have used animal models [13,14] and human granulomas [15,16] to investigate immune responses in TB infections. However, a comprehensive understanding of granulomatous tissues associated with cancer is lacking, and the role of PD-1 in interrogating cell phenotypes within the TME and whether changes in the TME correspond to changes in the TB granulomas (tuberculoma) of cancer patients have not been explored. This is due to the rarity of concurrently sampled multiple tissue types from a single patient with both cancer and TB reactivation. In a recent study, multiple distinct TMEs within individuals with cancer were found to be associated with different outcomes of metastatic lesions following therapy [17]. In this study, we utilize multiplexed immunohistochemistry staining on cross-tissues from two individuals with metastatic NPC and gastrointestinal TB reactivation during pembrolizumab treatment. Our aim is to characterize the tissue-specific immune heterogeneity in these patients and investigate whether heterogeneity is associated with treatment response. This study contributes to our understanding of tissue-specific immune microenvironments (TSIMEs) associated with ICI in benign, malignant and TB tissues. Through analysis of multiple tissue lesions, we have identified alternations in immune infiltrates, including subpopulation types, density, composition, functional orientation and spatial distribution. These changes potentially contribute to the development and persistence of aggressive anti-PD-1-related TB features in NPC patients undergoing ICI treatment. 

## 2. Materials and Methods

### 2.1. Study Design

This was an observational study on the detailed mapping of the immune cells in the tissues of two NPC patients (one was in their 20s, and one was in their 60s; details summarized in online supplementary information) with reactivation of TB after pembrolizumab treatment, managed at the Queen Mary Hospital, Hong Kong. They were retrospectively and sequentially recruited. Both provided written informed consents. The study was approved by the Institutional Review Board of The University of Hong Kong/Hospital Authority Hong Kong West Cluster (UW 19-157). 

Our study used serial sections as input for hematoxylin and eosin (H&E) and mIHC. Twenty-four protein markers were used to identify a total of 61 biomarker combinations (cancer cells, *n* = 3; immune cells, *n* = 48; 7 combinations identified for cytokine patterns and 3 identified for co-inhibitory/co-stimulatory receptor; refer to online supplementary Table S1 for protein markers, Supplementary Table S2 for immune cell lineages and identification and Supplementary Figure S2B for overall biomarker combination patterns). Four different multiplex panels were first used as an exploratory study including lymphoid, myeloid lineages, oncogenic and functional panels. In the exploratory study, twenty-six distinct immune cells were identified. One multiplex panel was used as a refinement study (molecular panel) with fifteen distinct immune cells identified. Using the mIHC data, we derived immune subset density (cells per mm^2^) and spatial characteristics (median cell-to-cell distance) to correlate with tumor microenvironment and the granuloma-associated immune cell microenvironment. All clinical details for the specimens are summarized in Supplementary Table S4. Please see Supplementary Materials for tissue collection details.

### 2.2. Patient Characteristics

The 29–year-old woman was diagnosed with metastatic NPC to her neck lymph nodes, lungs, and bones. Despite chemoradiation therapy, her disease progressed, and she was given pembrolizumab, with partial responses. Histologic image of the nasopharynx tumor revealed high expression of PD-L1, and she was given pembrolizumab. She developed tuberculosis in the ileum 21 months after ICI. After completion of anti-TB medication, histologic examination of the nasopharynx was benign. The terminal ileum and cecum showed no granulomatous inflammation. The second patient of a 65–year old man was diagnosed to have bulky stage II NPC. He received induction, followed by radical chemoradiation. He developed liver, lung, mediastinal and abdominal nodal metastases three years later. Six months after receiving pembrolizumab, the resected liver metastasis showed granulomatous inflammation Acid-fast bacilli could be detected in the granuloma of the falciform ligament and the duodenal lymph nodes. He then received anti-TB treatment for the next 12 months, while his pembrolizumab treatment was continued during this period. He further developed lung metastasis. 

### 2.3. Tissue Collection 

Eleven diagnostic specimens across different anatomic origins were considered: nasopharynx (*n* = 4), terminal ileum (*n* = 2), cecum (*n* = 1), falciform ligament (*n* = 1), duodenal lymph node (*n* = 1), lung (*n* = 1) and liver (*n* = 1). The specimens were collected during routine diagnostic and therapeutic measures. They were processed into formalin-fixed and paraffin-embedded (FFPE) blocks in a CAP-accredited service laboratory, according to the routine standard operation protocols. Serial sections (5 μm) of each specimen were stained with H&E and examined by an anatomic pathologist to screen for the presence of granulomatous inflammation and malignant cells. The consecutive tissue sections from each specimen were deparaffinized and rehydrated by serial passage through changes of xylene and graded ethanol for multiplex immunohistochemistry staining.

### 2.4. Image Capturing 

A total of 55 slides (5 panels × 11 slides) were imaged on the Vectra^®^ Polaris™ 3.0 Automated Quantitative Pathology Imaging System (Perkin Elmer, Waltham, MA, USA). Initially, whole slide scans were performed using a 20× objective lens with 7 standard epi-fluorescence filters (DAPI, Opal 480/520/570/620/690 and 780). Then, when the regions of interest (ROI; image size of 0.93 mm × 0.7 mm; resolution of 0.497 µm per pixel) was selected in the Phenochart program (Phenochart 1.0.9, Perkin Elmer), and images were recaptured with the 9-colour unmixing filters (DAPI, Opal 540/650, Opal 480, 520, 570, 620, 690 and 780). The ROIs of granuloma and malignant samples were confirmed with a clinical pathologist. The ROIs of non-malignant samples were randomly picked by two observers. All the settings applied to the training images were saved within an algorithm to allow batch analysis of multiple multispectral images of different tissues.

### 2.5. Quantification and Multiplexed Analysis

The analysis pipeline on inForm^®^ v2.4 (Perkin Elmer) started with the deconvolution of fluors, tissue and cell segmentation and cell phenotyping. Library slides were generated from representative NPC tissue sections to allow for accurate unmixing of the multiplexed samples, including a slide stained for each single fluorophore, a DAPI only slide and an autofluorescence slide wherein there was no antibody, Opal reagent or DAPI was applied. The unmixing performance of this tissue-specific spectral library was compared to that of the synthetic Opal library (built on tonsil) available in inform. Multispectral images were unmixed using our in-house spectra library built using the inform Advanced Image Analysis software (Perkin Elmer, Inc, Waltham, MA, USA, Ver.1.0.9). 

After deconvoluting the RGB images, tissue segmentation was performed with inForm^®^ Software (Version 2.4, Perkin Elmer, MA, USA). The tissue and background regions were indicated by the observer’s annotation, and tissue masks were generated to segment the tissue regions from the background. This was done to obstruct the nonessential calculation on non-tissue regions of the ROIs. Single cell segmentation was performed based on all cells counter-stained with DAPI, and the detection of fluorescence threshold was adjusted by the users on a batch-to-batch basis to accurately detect the number of cells. The quantification of each biomarker was aided by a machine learning algorithm, and the algorithm was trained based on the staining intensity and pattern with at least 5 cells indicated by the observer. The similarity of the phenotyping patterns between the observer’s annotations and each cell was represented as the confidence level. The quantification pattern of each biomarker was checked by two separate scientists. Density of cells in each ROI was calculated by combining the cell counts from all images and normalizing by the total area (number of cells/mm^2^). The quantification of each biomarker was aided by a machine learning algorithm, and the algorithm was trained based on the staining intensity and morphology. The similarity of the phenotyping patterns between the user’s annotations and each cell was set as 70% cut-off confidence level to ensure accurate quantifications of each cell type. The quantification pattern of each biomarker was further checked by two separate scientists. An R program was built to generate the cell profile by overlaying the single biomarkers based on the x and y coordinates of the cell location. The representative images were processed in the Quantitative Pathology & Bioimage Analysis software (QuPath 3.0). 

### 2.6. PBMC Cell Isolation from Human Blood

Human peripheral mononuclear cells (PBMC) were isolated from healthy donors (buffy coats, Hong Kong Red Cross Blood Transfusion Service) for subsequent culture. PBMC were isolated from healthy donors by Ficoll-Paque gradient (GE Healthcare) and centrifuged at 400× *g* for 25 min. The interface containing PBMC was carefully removed and cells were washed twice with PBS + 2 mM EDTA. After washing, PBMCs aliquots of 2 × 107 cells/vial were cryopreserved in 10% DMSO-containing media for extended storage in liquid nitrogen. When needed, PBMC vials were thawed and then washed in RPMI containing 5% human serum. Cells were suspended in RPMI containing 20% of heat-inactivated type AB human serum (P40-2701, PAN Biotech, Aidenbach, Bavaria, Germany) and counted by trypan blue dye exclusion method.

### 2.7. PBMC Infection and Three-Dimensional In Vitro Granuloma Formation

Human PBMC were thawed one day prior to assay. The day after, an extracellular matrix (ECM) was prepared by mixing 150 µL of 10× PBS (D1408, Sigma-Aldrich, St. Louis, MO, USA), 900 µL of type I collagen solution (3 mg/mL) (5005, Advanced BioMatrix, San Diego, CA, USA), 6 µL of 0.1% fibronectin (F0985, Sigma Aldrich, St. Louis, MO, USA) and 423 µL of Milli-Q water per 1.5 mL of matrix solution and kept on ice until use. Immediately before use, 21 µL of 1 M NaOH (pH 7.4) was added and mixed slowly, and the final pH of the solution should be ~7.0. Afterwards, PBMC were mixed at room temperature with ECM mixture at 2.5 × 106 cells/250 µL well of a 96-well plate. Ten µg/mL of recombinant Mtb CFP10: ESAT6 chimera protein (DAGA-193, Creative Diagnostics, Shirley, New York, NY, USA) was added to the ECM for sample infection with 25 mM HEPES, 2 mM of L-glutamine and 20% of heat-inactivated type AB human serum at 37 °C. The formation of granulomas was evaluated after 2 and 5 days of culture and captured using IN Cell Analyzer 6500 HS (GE Healthcare Life Science, Piscataway, NJ, USA). Only multilayered structures containing ~4–8 cell layers were considered granulomas. On day 5, granulomas were collected with manual pipette and incubated with 0.05% Trypsine-0.53 mM EDTA for 15 min at 37 °C to dissociate cells. Full culture details are provided in the online Supplementary Materials.

### 2.8. Flow Cytometric Analysis

Cells dissociated from granuloma were collected with manual pipette, incubated with 0.05% Trypsine-0.53 mM EDTA for 15 min at 37 °C to dissociate cells. Cells from five similar wells were pooled, washed and re-suspended in FACS buffer (PBS, 0.5% BSA, and 2 mM EDTA). Cells were then labeled with a mixture of the following fluorescent antibodies: CD45-PerCp CD8-APC-Cy7, CD137-Alexa700 and PD-1-FITC(all from BD bioscience. San Jose, CA, USA). Cells were analyzed on a NovoCyte Quanteon and the data were analyzed using FlowJo Software (TreeStar Ashland, OR, USA, Ver10.7.2).

### 2.9. Whole-Blood and Tumor Tissue Transcriptomic Analysis

Estimation of cellular abundances was performed based on the Gene Expression Omnibus (GEO) repository via CIBERSORT. Data from publicly available microarray cohorts related to gene expression in TB patients (GSE28623, GSE62525, GSE81746) to assess gene expression differences across clinical groups (healthy and latent TB) and RNA tumor tissues sequencing data related to gene expression associated with PD-1 drug responsiveness in NPC patients (GSE136961) were downloaded from the GEO repository. CIBERSORT L22 reference expression and modified expression in genes of interest were used as the expression signature for cell abundance analysis using CIBERSORT (https://cibersort.stanford.edu/ (accessed on 9 May 2022)). Abundance ratio relative to healthy individual in TB cohorts and relative to non-durable benefit in NPC cohort was calculated for comparison. The adjusted p-value was calculated using ANOVA in GraphPad Prism (Version 6).

### 2.10. Statistical Analysis

GraphPad Prism 6 or SPICE 6 or R.3.5.1 and R studio 1.1.456 were used to generate quantitative graphical representation of the generated data and statistical tests. Schematic visualizations were produced with Biorender. Log 2 transformation was done when appropriate, and all the 0 values were treated as 0.1 for computational reasons. The Mann–Whitney U test was used for comparing two study groups for assessing the statistical significance. The comparison between three samples (G, M and N-DP) was conducted with Dunn’s test. T-distributed stochastic neighbor embedding (t-SNE) analysis was done based on the cell density or the single cell fluorescence signal levels for each Opal dye. Different clustering methods were used to determine the optimal number and pattern of clusters. K-mean clustering was used for clustering of at least 4 samples, and hierarchical clustering method was used for clustering of less than 4 samples. The perplexity level was also adjusted for optimizing the clustering pattern. The data input for the interactive network analysis was binary. The existence of interaction between two cell types was represented as 1, and the lack of interaction was represented as 0. The distance analysis was conducted with the R package “phenopt”, and it was based on identifying the mutual closest distance between two cell types.

### 2.11. Multiplexed Tissue Staining

FFPE slides underwent hexa-, hepta- and nano-color multiplex immunofluorescence staining kits (Akoya Biosciences, Marlborough, MA, USA) according to the manufacturer’s instructions. In brief, the oncogenic panel included DAPI, LMP1, PD-L1, PD-1, IFNγ and pan-cytokeratin (tumor marker); the myeloid panel included DEC205 (dendritic cells), PD-L1, MHCI, MHCII, CD83, CD163 (type 2 macrophages, M2) and CD68 (type 1 macrophages, M1); the lymphoid panel included CD4 (T-helper), CD8 (cytotoxic-like), T-bet (T-helper 1), GATA3 (T-helper 2), RORγt (T-helper 17), FOXP3 (T-regulatory) and PD-1; the functional panel included CD4, CD8, Ki67, PD-1, Eomes, IFNγ, T-bet and TNFα; the cellular cytokine panel included CD8, CD137, DEC205, IL12, CD163, IFNγ, CCL17 and CD68. An example of the experimental pipeline is shown in Figure S1A, and quality control data are shown in Figure S1B–D. For information on antibodies and specific staining conditions, see Tables S3 and S5–S7. Biomarkers with inferred phenotypes are outlined in Table S2. 

### 2.12. RNA-Sequencing and Microarray Analysis of Data from Public Databases

To estimate cellular abundances and interactions from the Gene Expression Omnibus (GEO) repository via CIBERSORT, data from publicly available microarray cohorts related to gene expression in TB patients (GSE28623, GSE62525, GSE81746), and RNA sequencing data and microarray cohorts related to gene expression related to PD-1 drug responsiveness in cancer patients (GSE13691, GSE93157) were downloaded from the GEO repository. 

### 2.13. Data Analysis, Bioinformatics and Statistical Considerations

Full details for data analyses, statistical tests and power calculations are provided in Table S16.

## 3. Results

### 3.1. Characteristics of Two NPC Patients with Reactivation of Gastrointestinal TB after Pem-Brolizumab Treatment

**Figure 1:**
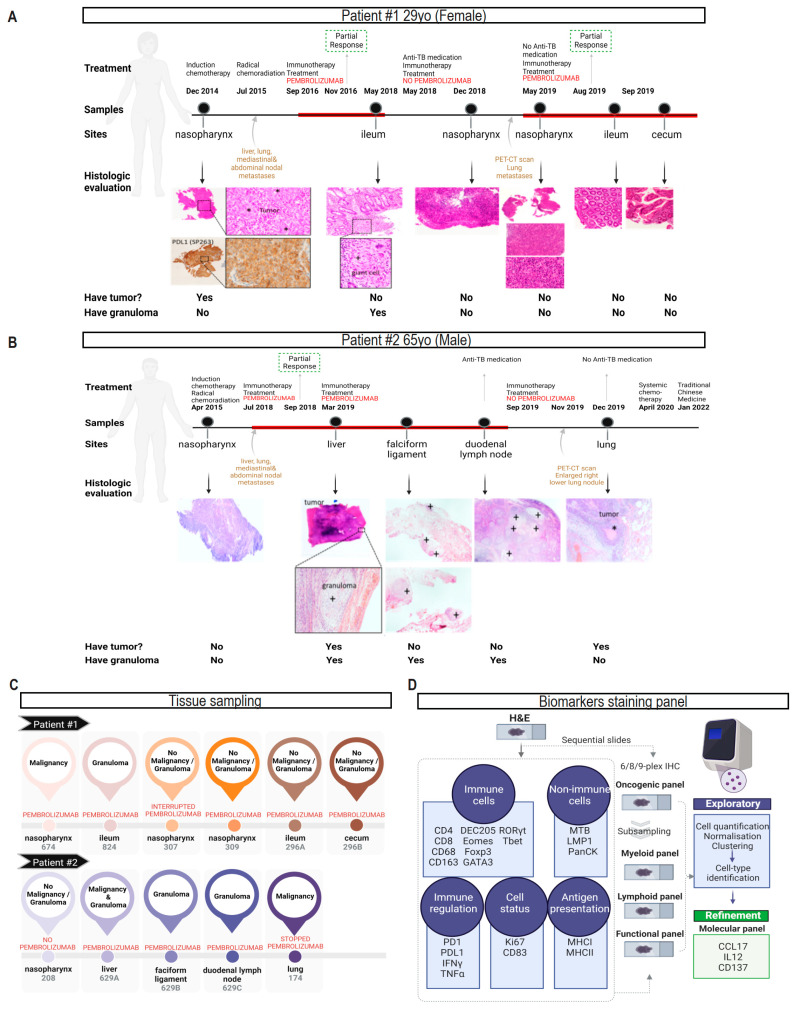
Workflow for data integration on TB development after PD-1 blockade in two NPC patients. (**A**) Histologic image of the nasopharynx tumor of Patient #1 (H&E staining; crosses (+) indicate region with tumor) show high expression of PD-L1 (DAB peroxidase staining; inset shows a high-power view). High-power view of the histologic section of the ileum (crosses (+) indicate giant cells) shows granuloma in mucosa. Red line indicates treatment period on pembrolizumab; yo, years old. (**B**) Histologic image of the resected liver metastasis from Patient #2 showed granulomatous inflammation (crosses (+)) adjacent to the tumor (asterisks (∗)). Acid-fast bacilli could be detected in the granuloma of the falciform ligament and the duodenal lymph nodes. Red line indicates treatment period on pembrolizumab; yo, years old. (**C**) Eleven diagnostic specimens across different anatomic origins of the two patients were available for analysis. These specimens were broadly categorized according to the presence or absence of tumor or granuloma. The wedge excision of the liver metastasis of Patient #2 was assigned as two discriminate components for tumor and granuloma, respectively. (**D**) Experimental approach for the twenty-four biomarkers and five panels for multiplex staining.

In the first case, a patient in their 20s with metastatic NPC to neck lymph nodes, lungs and bones developed TB in the ileum 21 months after ICI treatment (Figure 1A) [6]. In the second case, a patient in their 60s with bulky stage II NPC, who had no previous TB infection, showed granulomatous inflammation in the adjacent liver tissue six months after receiving pembrolizumab (Figure 1B). To analyze cellular heterogeneity, we utilized multiplexed immunohistochemistry (mIHC) with data acquisition and processing for 11 biological tissues (Figure 1C, Figures S1 and S2A), allowing us to study 61 combinations of 24 biomarkers in our cross-tissue observational study. (Figure S2B; Tables S1 and S2). 

### 3.2. Clustering Samples Based on Intrinsic Shared Biological Features of Benign, Malignant and Tuberculosis Tissue Lesions

Initially, we compared the two patients independently but faced challenges due to the lack of matched pre- and post-ICI clinical samples and the composition of samples acquired from multiple anatomical sites at different time points. To overcome inter- and intra-patient tissue heterogeneity, we developed a staining panel and implemented standardized analysis pipelines to eliminate unwanted variation. In EBV-positive NPC, immune resistance attributed to PD-L1 expression can be regulated by oncogenic pathways like LMP-1 and inflammatory signals such as interferon-γ (IFNγ) [18]. While IFNγ serves as a pro-inflammatory marker for TB [19], it alone is not sufficient to trigger TB reactivation or activation; it requires PD-1 depletion in the tumor [13]. Considering these factors, we included five markers in our IHC panel: pan-cytokeratin (PanCK, a marker for epithelial cells), LMP1, IFNγ, PD-L1 and PD-1, to analyze intrinsic cross-tissue biological characteristics across multiple samples from different tissues and individuals (Figure 1D; Table S3). 

**Figure 2:**
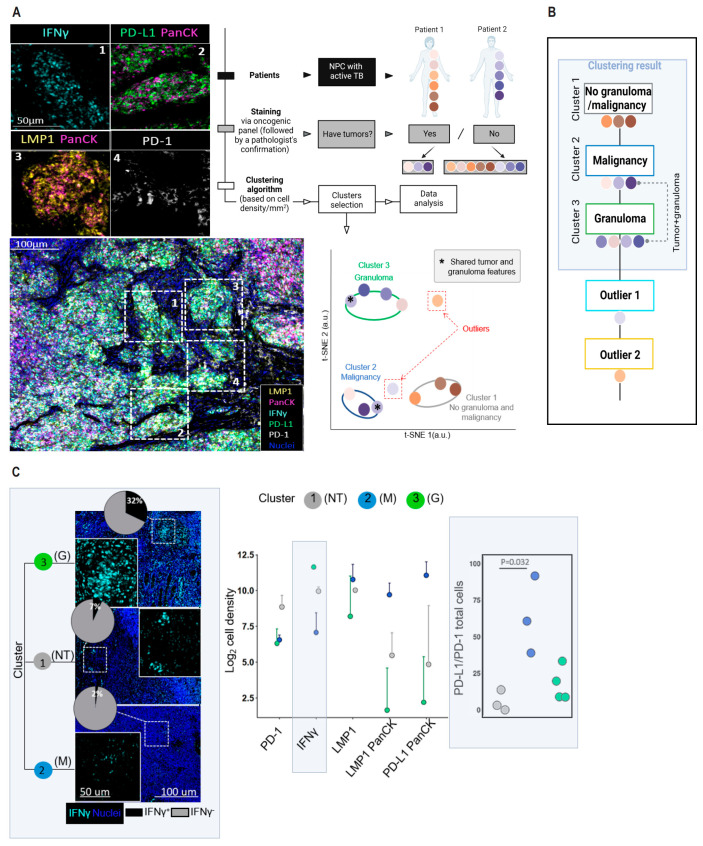
Classification of tissue clusters and identification of IFNγ-enriched granuloma. (**A**) NPC cohort of specimens and schema used in this study. Zoom images of the area as indicated by the square labelled with 1–4. The samples were stained with 5 biomarkers. The color overlay of IFNγ+ cells (magenta), PanCK+ epithelial cells (pink), PD-L1 (green) and LMP-1 (yellow) is shown in a representative region of the nasopharyngeal tumor region. The tissues were clustered into a scatter plot, with three clusters (cluster 1 in grey, *n* = 3; cluster 2 in blue, *n* = 3; and cluster 3 in green, *n* = 4) and two tissue outliers (denoted inside dotted square in red) identified. The two outliers were from NT samples collected pre-medication (NT-P; outlier 1) and during treatment interruption (NT-I; outlier 2). One sample was grouped into cluster 2 and 3 individually due to sharing tumorous and granulomatous features (indicated with “∗”). (**B**) Samples were generated by subsampling. (**C**) Scatter plot of IFNγ log2 transformed cell density data among different cluster groups. Left: representative image of stained IFNγ immune cells (turquoise) in respective clusters are shown. The pie charts show the percentage of IFNγ+ cells. Samples showing no tumorous or granulomatous features are denoted as “NT”. Right: ratio of PDL1+ to PD1+ immune cells. Data information: data points are shown ± SEM. In (**A**,**C**), nuclei are counterstained with DAPI, shown in blue. Scale bars, 100 μm. Enlarged inserts: scale bars, 50 μm. In (**C**), *p* values were calculated by Dunn’s test with Bonferroni adjustment.

Using contemporary hierarchical methods based on cellularity, we classified tissue samples into tumor or non-tumor (NT) categories, resulting in three distinct tissue clusters: M (malignant), G (granulomatous) and NT, and two outliers (Figure 2A; Table S4). Importantly, all three clusters included samples from both patients, indicating similar immune signatures across patients in different tissues (Figure 2B).

### 3.3. Multi-Tissue mIHC Characterization of Immune Landscapes in Patient Tissues

Previous research has indicated that higher levels of IFNγ are linked to an increased risk of developing TB [20]. In our study, we found that the inflammatory IFNγ+ cells were predominantly expressed in cluster 3 (G) (3277.1 ± 449.4 cell/mm^2^), accounting for 32% of total cells. In contrast, there were only 7% in cluster 1 (NT) (1041.7 ± 224.5 cell/mm^2^) and 2% in cluster 2 (M) (318.4 ± 257.6 cell/mm^2^). Additionally, animal studies have shown that PD-1 knockout (KO) in mice facilitated granuloma formation [21,22]. We observed that PD-1 was less abundant in cluster 3 (G) and cluster 2 (M), where PD-L1+ cells outnumbered PD-1+ cells, compared to cluster 1(NT) (ratio (PD-L1+/PD-1+)  = 63.0 ± 15.3 for cluster 2 (M); 17.4 ± 5.8 for cluster 3 (G) and 5.6 ± 4.1 for cluster 1 (NT), respectively; Figure 2C). Our findings suggest that gastrointestinal TB infection is associated with a depletion of PD-1 and increased IFNγ production. 

### 3.4. CD8+ T Cells Dominate the T-Cell Infiltrates in the Different Tissue Types

We employed two biomarker panels, consisting of 13 markers, to classify lymphoid and myeloid lineage cells. The lymphoid panel identified various T cell subsets, including CD4+, CD8+, T-helper1 (Th1), T-helper2 (Th2), T-helper17 (Th17) and regulatory (Treg) T cells. The myeloid panel identified classical M1 macrophages (CD68+CD163-), alternative M2macrophages (CD68+CD163+) and dendritic cells (DEC205+, DCs) (Tables S5 and S6). 

Surprisingly, our analysis did not reveal a higher abundance of macrophages in cluster 3 (G) tissues, contrary to previous studies emphasizing their role in initiating inflammation within granulomas [23]. We also observed minimal variations in macrophage polarization across all tissue types (Figure S3A). Additionally, we characterized myeloid cell subpopulations based on activation (MHC class I/II, CD83) and immunoregulatory signals (PD-1 and PD-L1) (Figure S3B), finding only a slight increase in PD-L1 expression in cluster 2 (M) tissues, contradicting previous findings [15]. 

**Figure 3:**
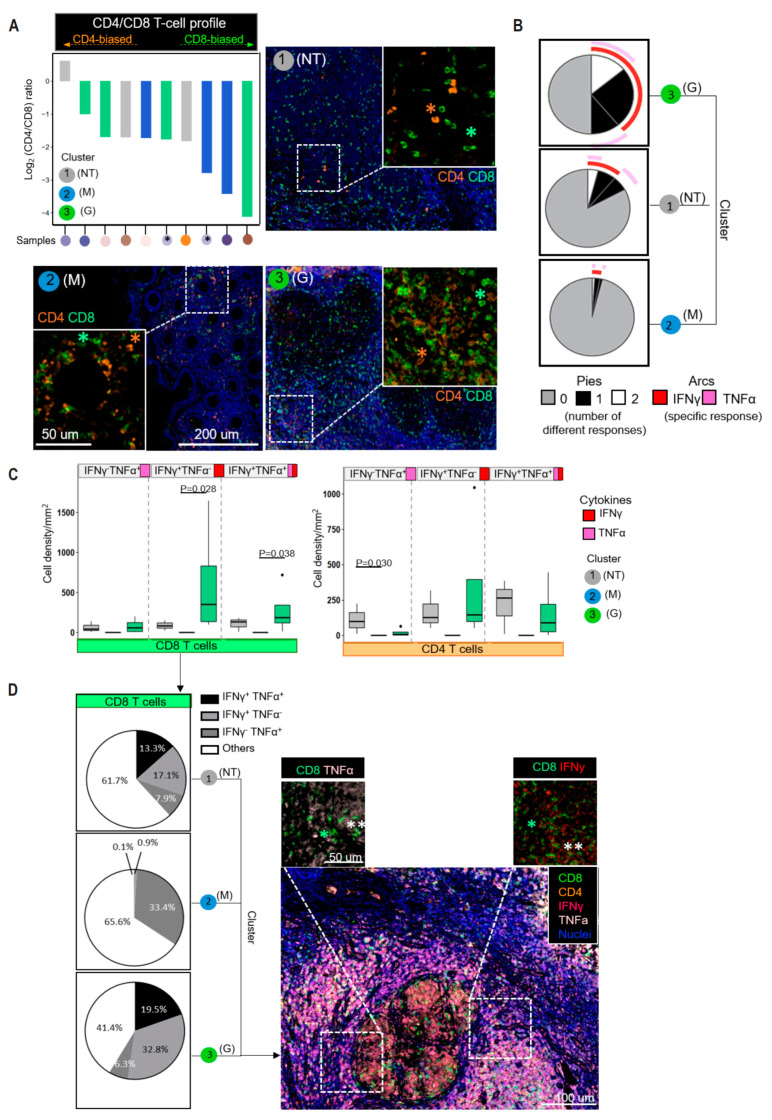
Tuberculoma highly enriched with IFNγ-releasing CD8+ T cells. (**A**) Representative images of mutually exclusive markers (CD4/CD8). Green: tissue with granulomatous features as cluster 3; blue: tissue with tumorous features as cluster 2; grey: non-tumorous as cluster 1. One sample grouped into cluster 2 and 3 individually due to sharing tumorous and granulomatous features (indicated with “∗”). Zoom-in is indicated by white dotted-line squares. Exemplary cells are annotated with respective color codes: orange asterisks (∗) as CD4 and green asterisks (∗) as CD8. The CD4+ T-cell/CD8+ T-cell ratio is represented as a log2 fold change for each specimen. (**B**) The segments within the pie chart denote the populations producing different combinations of cytokines and are color-coded (white–gray–black). Size of the pie segment correlates to the frequency of corresponding populations. Arcs around the circumference represent specific cytokine (TNFα in pink; IFNγ in red) produced by the proportion of cells that lie under the arc. Parts of the pie surrounded by multiple arcs represent polyfunctional cells. (**C**) Boxplots display TNFα- and/or IFNγ-producing CD4 and CD8 T cells (cell/mm2) in different clusters. The centerline indicates the median, while the upper and lower lines represent the 75th and 25th percentiles, respectively. (**D**) Pie charts represent average frequencies of CD8+ T cells producing combination of any two cytokines. Representative images of TNFα- and/or IFNγ-producing CD8+ T cells in granuloma. The boxes with dotted lines show the magnified areas. Exemplary cells are annotated with respective color-coded asterisks, where (∗) indicates a single-positive marker and (∗∗) identifies double-positive biomarkers..Data information: in (**A**,**D**) nuclei are counterstained with DAPI, shown in blue. In (**A**), top panel: scale bars, 200 μm; lower panel: scale bars, 50 μm. In (**D**), scale bars, 100 μm; enlarged inserts: scale bars, 50 μm. Outliers are represented by individual points. *p* values were calculated by Dunn’s test with Bonferroni adjustment.

The balance of CD4+ Th cell subsets (Th1/Th2/Th17/Treg) is crucial in determining the outcome of MTB infections [24]. To understand this, we examined the expressions of transcription factors (T-bet, GATA3, RORγt and FOXP3) that regulate the development of these subsets [25]. However, we did not find an enrichment of CD4+ Th lineages in either cluster 2 (M) or cluster 3 (G) tissues in our dataset (Figure S3C). Therefore, we cannot conclude whether an imbalance of Th1/Th17 responses with exaggerated Th2/Treg confers susceptibility to MTB infection in our study, which contrasts with a previous report [22]. It is worth noting that CD4:CD8 T cell ratios predominantly favoring CD8+ T cells were observed in the granulomatous lesion, consistent with a previous report [15]. In particularly, cluster 2 (M), cluster 3 (G) and cluster 1 (NT) have the highest to lowest proportion CD8+ T cells, respectively (Figure 3A; log2(CD4+:CD8+ T cell ratio) = −2.64 ± 0.49 for cluster 2 (M); −2.14 ± 0.68 for cluster 3 (G); −0.97 ± 0.79 for cluster 1 (NT), respectively). The finding that distinct CD4:CD8 T cell ratio is exhibited in different pathological lesions, despite CD8 T-cell immunodominance, suggests the existence of a unique TSIME for each lesion upon ICI treatment.

### 3.5. IFNγ-Producing CD8+ T Cells Significantly Accumulate in the Granulomatous Tissues

Previous studies have linked decreased frequencies of Ki67+ Eomes+ CD4+ T cells with irAE [26], while increased levels of Ki67+ PD1+ CD8+ T cells are linked to prolonged progression-free survival in melanoma patients treated with anti-PD-1 therapy [27]. We aimed to determine if T cells are locally proliferating or have different activation phenotypes (Table S7) and found a strong depletion or enrichment of Ki67+ CD8+ T cells and Ki67+ CD4+ T cells in cluster 2 (M) and cluster 3 (G), respectively (Figure S4A,B), indicating T-cell proliferation/reinvigoration in the PD-1-induced tuberculoma. Furthermore, we noticed a trend of decreased frequencies of Ki67+Eomes+ and Ki67+PD1+ T cell subsets (CD8+ and CD4+) in cluster 2 (M) and cluster 3 (G), respectively. Although these findings did not reach statistical significance, they suggest that the presence of Ki67+Eomes+ and Ki67+PD1+ T cell subsets in the TME of NPC may suggest susceptibility to irAE. 

To determine T-cell activation, we assessed key cytokine production such as IFNγ and tumor necrosis factor α (TNFα). Our analysis found that TNFα- and/or IFNγ-producing cells were less dense in cluster 2 (M) tissues. In contrast, cluster 3 (G) tissues had the highest proportion of TNFα (11%), IFNγ (25%) and dual TNFα-IFNγ producers (14%) (Figure 3B). When we looked at T-cell subsets, a higher proportion of CD4+ T cells from cluster 1 (NT) expressed TNFα, while CD8+ T cells expressing IFNγ were strongly enriched in cluster 3 (G) tissues (mean TNFα+CD4+ T cell density: cluster 1 (NT), 111.8 ± 106.8; cluster 2 (M), 0; cluster 3 (G), 20.2 ± 30.0; mean IFNγ +CD8+ T cell density: cluster 1 (NT), 88.5 ± 64.6; cluster 2 (M), 4.6 ± 5.5; cluster 3 (G) 613.4 ± 720.0; Figure 3C,D). The CD8+ T-cell subsets in cluster 3 (G) had a higher proportion of IFNγ than TNFα-IFNγ double producers (mean TNFα-IFNγ+CD8+ T cell density: cluster 2 (M), 4.6 ± 3.2; cluster 3 (G), 613.4 ± 359.7; mean TNFα+IFNγ+CD8+ T cell density: cluster 2 (M), 0.5; cluster 3 (G), 278.2), suggesting that CD8+ T cells with high IFNγ production may be driving granulomatous inflammation.

### 3.6. Infiltrating Immune Cells Are Phenotypically and Functionally Heterogeneous in Different Tissue Types

**Figure 4:**
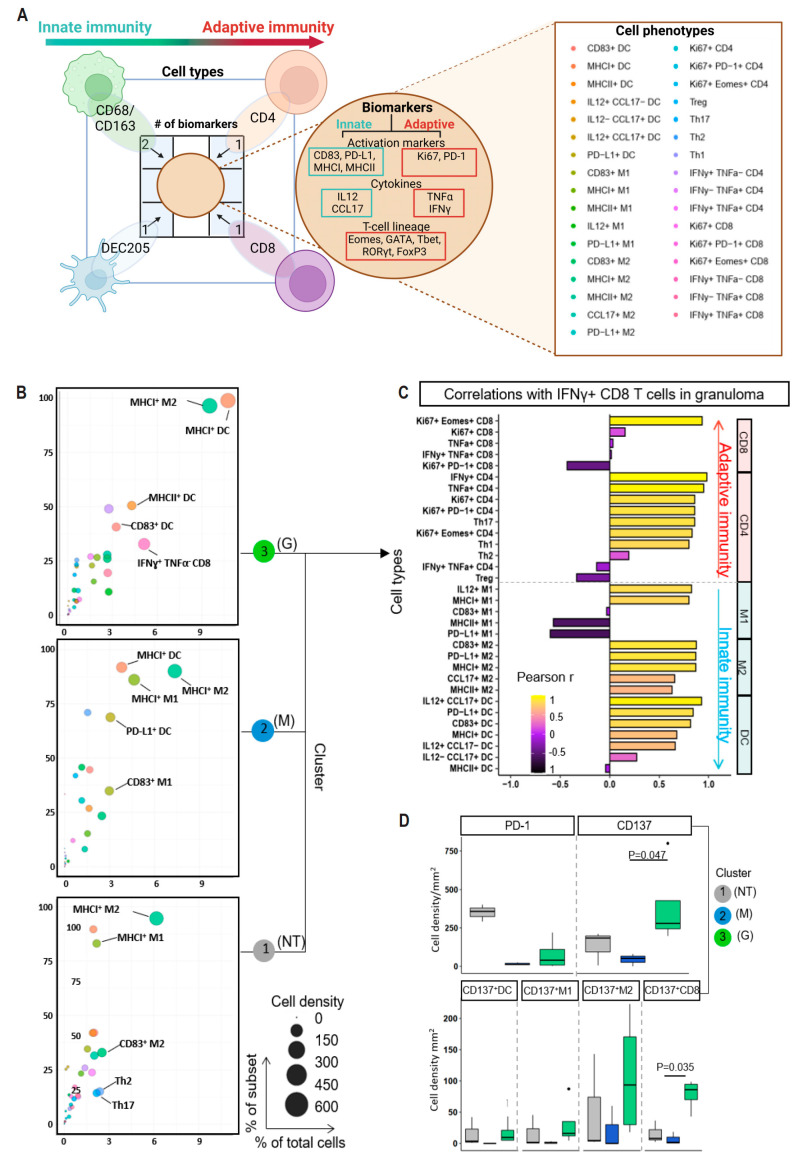
Heterogeneous structured immune microenvironments across different tissue types. (**A**) Schematic representation of single, double and triple positive cells in myeloid (teal) and lymphoid (red) linages. Twenty biomarkers were used to identify 33 cellular subsets. (**B**) Bubble-chart plots summarizing frequencies of all cell types among all tissue groups. The top five ranked cells are indicated. The bubble size was proportional to the cell density. X-axis represents percentage of indicated cellular subset in total cells, and y-axis indicates cellular subset in myeloid-/lymphoid linage. All the different cellular subsets are color coded. (**C**) Pearson’s pairwise correlation among cell type proportions in relation to IFNγ+ CD8 T cells in granuloma. (**D**) **Top**: expression levels of PD1 and CD137. **Bottom**: CD137-expressing immune cells. Individual points are outliers.

In the tuberculoma niche, the coordinated expression of cytokine is crucial [28]. To further explore our above findings, we investigated the cytokine/chemokine profiles that could determine M1/M2 polarization [29]. Due to limitations in multiplexing capacity, we focused on two representative cytokines, IL12 and CCL17. These biomarkers not only delineate myeloid compartments (M1, M2 and DC), but also play a role in inflammatory responses, CD8+ T-cell responses, and maintenance and regulation of granulomatous responses [30,31,32]. In total, we analyzed 20 immune biomarkers to identify 33 immune cell types across different tissues (Figure 4A; Table S8). 

Quantitative analysis of fluorescent multiplex immunohistochemistry staining revealed unique immune cellularity in TSIMEs across two patients (Figure 4B; Tables S11–S13 depict cell proportion and cell density; Table S14 depicts mean and range of cell density). Cluster 3 (G) had a significant enrichment in total immune cell abundance compared to other tissues, while interrupting pembrolizumab led to a decrease in immune cells in NT tissues (i.e., NT-I, outlier 2), and an increase in NT-P (outlier 1) (Figure S5, Tables S9, S10 and S15). Among the top five ranked cell types, myeloid cells, particularly MHCI+ M1 or MHCII+ M2, were commonly and heavily accumulated across all tissue clusters (i.e., MHCI+ M1 or MHCII+ M2), while IFNγ+CD8+ T cells were the most abundant adaptive cell type found in tuberculoma (5.1% of granuloma cells). We speculated that the enriched IFNγ+CD8+ T cells may associate with other immune cells to facilitate the development of PD1-related tuberculoma. To investigate this, we calculated Pearson correlations between the densities of 32 cell types/subpopulations and IFNγ+CD8+ T cells within the tuberculoma niche (Figure 4C). We found several innate cell types positively associated with IFNγ+CD8+ T-cells, including IL12–producing M1 and IL12/CCL17-coexpressing DC.

### 3.7. Enrichment of CD137 in Granuloma-Residing CD8+ T Cells

Previous studies have demonstrated that CD137 stimulation induces the production of IFNγ by CD8+ T cells [33], and chronic signaling activation of CD137 has been linked to the development of granuloma [34]. In line with this, CD137-expressing cells, particularly, CD137+CD8+ T cells were found to be highly accumulated in cluster 3 (G) (Figure 4D; mean CD137+ cell density: cluster 1 (NT), 134.4 ± 102.5; cluster 2 (M), 44.9 ± 41.4; cluster 3 (G), 390.6 ± 276.4; mean CD137+ CD8+ cell density: cluster 1 (NT), 15.9 ± 14.2; cluster 2 (M), 6.7 ± 10.2; cluster 3 (G), 78.5 ± 25.1). Among all the examined innate cell types (i.e., M1, M2 and DC cells), CD137 expression on DC cells was less prevalent in the tuberculoma niche than in others. Additionally, IL12 and CCL17-expressing cells were enriched in the cluster 3 (G) tissues (mean IL12+ cell density: cluster 1 (NT), 203.2 ± 1765.5; cluster 2 (M), 308.3 ± 247.7; cluster 3 (G), 1032.0 ± 879.8); mean CCL17+ cell density: cluster 1 NT, 355.2 ± 256.6; cluster 2 (M), 279.8 ± 220.6; cluster 3 (G), 461.8 ± 351.3; Figure S6A). Interestingly, our correlation analysis revealed a negative association between PD-1 and IFNγ/IL12 (correlation index: −0.59 and −0.45, respectively; Figure S6B), while a positive association was observed between CD137 and IFNγ/IL12/CCL17 (correlation index = 0.86, 0.93 and 0.99, respectively). These findings suggest that inflammatory cues involving CD137/IFNγ/IL12/CCL17 are strongly presented in PD1-driven tuberculoma, which are less prevalent in non-cancerous tissues and even weaker in the tumor.

### 3.8. Spatial Proximity of CD137-Expressing Immune Cells in the Reactivation of TB Granuloma

**Figure 5:**
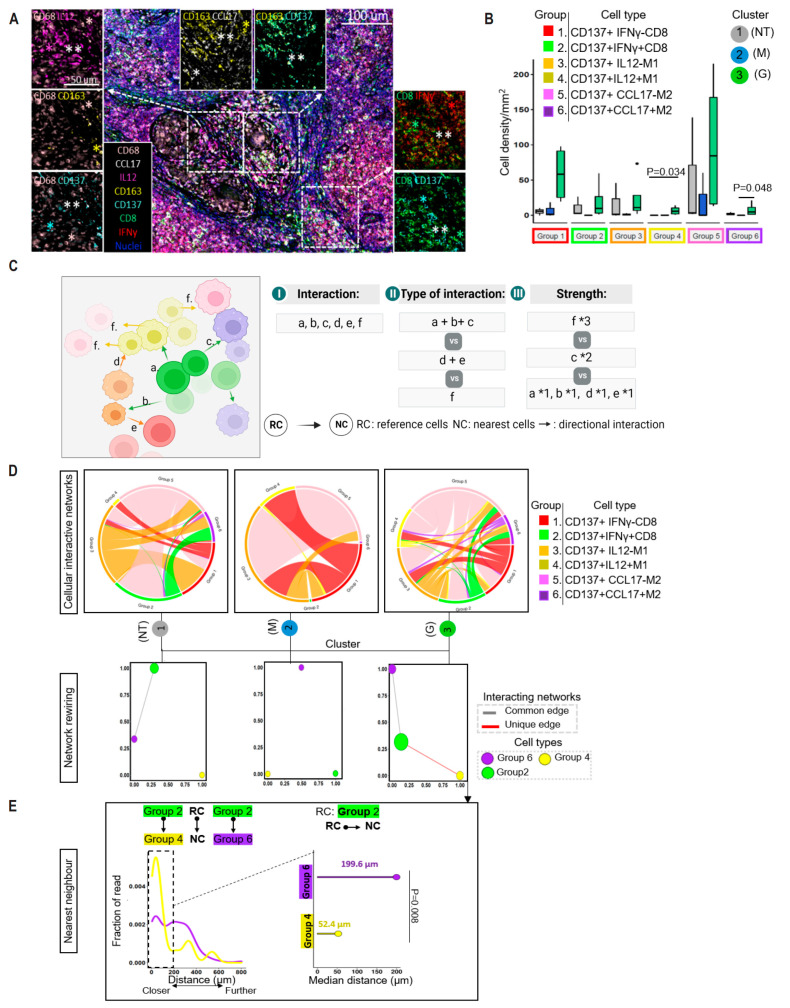
Distinct cellular interacting networks in granuloma. (**A**) Representative images of a granuloma. Nuclei are counterstained with DAPI, shown in blue. Scale bars, 100 μm; enlarged inserts: scale bars, 50 μm. Zoom-in is indicated by white dotted-line squares. Exemplary cells are annotated with respective color codes: single asterisks (∗) as single positive for respective marker and double asterisks (∗∗) as double-positive respective markers. (**B**) Boxplots show composition of each tissue types by CD137+ cell type groups. Individual points are outliers. (**C**) Schematic representation of the cellular interactive networks. (**D**) **Top**: network of interactions across cell type groups, subsetted to interactions strengthened across tissue clusters. Arc length of each segment corresponds to the relative frequency of each cell type group. Within each plot, interaction network pairs are represented by colored ribbons (type of interaction). Ribbon width proportional to number of interactions between represented cell type groups (strength of interaction). **Bottom**: spatial rewiring network. Nodes in the network represent unique cell type, and node sizes are proportional to the densities of respective cell types. Edges represent spatial co-expression between cell types. Red line indicates unique connection. (**E**) Histograms show all (**left**) or given distance (**right**) in μm between reference cell (RC) type group and nearest cell (NC) type group within granuloma. Solid dot represents RC, and arrow line represents NC. The dashed rectangle highlights the most differential distribution between respective RC to NC. Median value of the nearest distances (range from 0 to 200 µm) indicated in the plot. *p* values were calculated with a Wilcoxon test.

To examine the impact of CD137-associated tissue features on immune response, we compared different cell types expressing CD137, grouped as 1–6, based on their production of IFNγ, IL12 and CCL17 among CD8+ T cells and M1 and M2 cells (Figure 5A). Our findings showed that tuberculoma had higher densities of group 4 (CD137+ IL12+ M1 cells) and group 6 (CD137+ CCL17+ M2 cells) cells compared to other tissues (Figure 5B; mean group 4 cell density: cluster 1 (NT): 0, cluster 2 (M): 0.3 ± 0.4 and cluster 3 (G): 6.6 ± 6.1; mean group 6 cell density: cluster 1 (NT): 2.2 ± 1.8, cluster 2 (M): 0 and cluster 3 (G): 7.9 ± 9.4). Additionally, the frequencies of group 2 cells (CD137+ IFNγ+ CD8+ T cells) were higher in cluster 3 (G) tissues compared to cluster 2 (M) tissues (marginally significant, *p* = 0.06).

We also analyzed the spatial interactions between these cell types within each TSIME (Figure 5C). Cluster 3 (G) tissues displayed the most prevalent interactions between innate and adaptive cells (Figure 5D), including a unique interaction between group 2 and group 4 cells. This interaction was lacking in cluster 2 (M) tissues. Further analysis, using a network directed rewiring model [35], revealed networks stemming from group 2 cells and terminating in group 6 cells in both cluster 2 (NT) and cluster 3 (G) tissues, while networks stemming from group 2 cells and terminating in group 4 cells were only observed in cluster 3 (G) tissues. We further conducted a spatial distance analysis on those dual networks stemming from group 2 cells in the tuberculoma. A majority of group 2 cells were found to interact with group 4 cells (~73.3% (median: 53.3%, SD: 40.8%) RC- to -NC over total RC) where group 2-group 4 cells were located in closer proximity than group 2-group 6 cells (median value of the nearest distance: group 2- group 4: 52.4 ± 158.1 µm and group 2-group 6: 199.6 ± 128.1 µm; Figure 5E). In contrast, non-cytokine-associated CD8+ T cells (i.e., group 1; CD137+IFNγ-CD8+ T cells) were located further from group 4 or 6 cell types (median nearest distance: group 1-group 4: 340.3 ± 300.3 µm; group 1-group 6: 146.1 ± 213.0 µm; Figure S6C). These findings suggest that the juxtacrine-type cell-cell interactions between IFNγ-producing CD8+ T cells and IL12-producing M1 cells are specific to anti-PD-1-related tuberculoma and depend on CD137 enrichment.

### 3.9. CD137-Rich Cellular Microenviornment That Is Spatially Organized in Tumor Tissues in Response to ICI Is Absent in Ordinary TB

**Figure 6:**
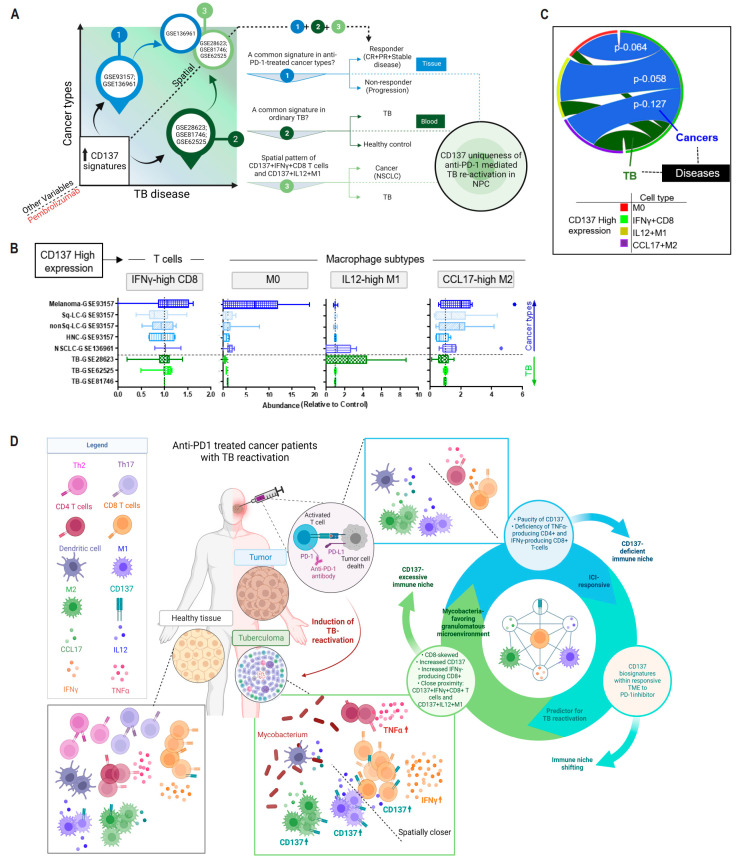
CD137+ cellular interaction is conserved in NPC patients responsive to anti-PD1 treatment. (**A**) Schematic approach. Publicly available microarray cohorts related to TB disease (GSE28623, GSE62525, GSE81746), and RNA sequencing data and microarray cohort related to anti-PD-1 drug responsiveness in cancer patients (GSE13691, GSE93157) were used. (**B**) Boxplots showing CD137 expression on four immune subsets across five types of cancer cohort tissue samples: melanoma, non-small-cell lung carcinoma (NSCLC), head and neck cancer (HNC), squamous lung cancer (Sq-LC) and nonsquamous lung cancer (nonSq-LC); and of three TB disease cohort blood samples. The median abundance level was used as the cutoff to divide the expression into high (hi) and low (lo) cell subsets. Individual points are outliers (**C**) Circos plot showing cellular interaction was estimated by correlations between cellular abundance in cancer (blue) or TB (green) disease. Adjusted p value was calculated using ANOVA. (**D**) Schematic model of spatial cellular heterogeneity in tissue zones of NPC patients with TB re-activation after anti-PD1 blockade. The image was created using BioRender.

We questioned whether CD137 signatures could be found in various types of human cancers after anti-PD-1 treatment, as well as in classical TB (i.e., TB unrelated to ICI treatment). We also explored whether the CD137-related intercellular interaction pattern is unique to ICI-related TB reactivation in NPC (Figure 6A). To explore these inquiries, we analyzed public databases to compare the levels of CD137 highly (CD137hi) expressing-IFNγ-producing CD8+ T cells, IL12-producing M1 cells and CCL17-producing M2 in tumor tissues from five different cancer types, depending on the response to anti-PD-1 treatment. Unfortunately, we were unable to obtain specimens of non-cancer related tuberculoma (classical TB) to study intra-granuloma features at a single-cell resolution. Therefore, we analyzed public whole blood transcriptomic datasets from patients with active TB as an alternative.

Our findings indicate that CD137hiIFNγhi CD8+ T cells are more prevalent in the TME of responsive cancer patients, although the extent of enrichment varies across different cancer types. We also observed higher CD137 expression in M0 and CCL17hi M2, but not in IL12hi M1 cells, indicating that increased expression of CD137 on tumor-infiltrating cells is associated with improved response to anti-PD-1 treatment, but this may be specific to certain cancer types. Comparing peripheral blood samples from healthy individuals and TB patients, we found a modestly higher abundance of CD137hiIFNγhi CD8+ T cells and CD137hiIL12hi M1 in TB patients (Figure 6B). This supports the hypothesis that niches with more CD137+ cells may have a greater tendency to develop into granulomas. We also compared intercellular interactions in a representative cancer type (non-small-cell lung carcinoma, NSCLC) and TB cohorts. We found a strong interaction between CD137hiIFNγhi CD8+ T-cells and CD137hiIL12hi M1 cells in NSCLC (with marginal statistical significance with *p* = 0.058; Figure 6C), but this interaction was weaker in TB disease. These results suggest that CD137+ T cell-macrophage interactions are enriched in the responsive TME to anti-PD-1 treatment. However, this interactive network is weakened in non-ICI-related TB disease, indicating that CD137+ T cell-macrophage-based intercellular communication may not be a general feature of all TB diseases. 

## 4. Discussion

Tumors’ development and response to anti-tumor therapies can be influenced by their heterogeneity and microenvironments [36]. Understanding the interaction between tumors and TSIMEs at different sites is challenging, and treatment-related immune-related adverse events can impact response. Additionally, we faced a common problem often met by biological researchers: the limited availability of samples for a focused disease. In this paper, we addressed this challenge by jointly analyzing multiple tissues from two NPC patients with TB reactivation after pembrolizumab treatment. Our study provides evidence of distinct TSIMEs within each patient, exhibiting similar immune signatures across patients. Despite limited specimens, we observed distinct treatment-associated immune cellularity that varied as tissue transitioned into a malignant or TB niche. This supports the clinical utility of histopathological screening to understand intra- and inter-TSIME which could potentially drive tumor progression, malignant transformation and ICI-associated adverse responses like TB reactivation.

In tumor tissues, we observed low levels of IFNγ and CD8+ cells, while the tuberculoma showed high levels of CD137+ cells and IFNγ-producing CD8+ T cells. This proof-of-concept study suggests that despite anti-PD-1 treatment, the tumor core remains immunosuppressive, increasing the risk of CD137-mediated immune-related adverse effects such as TB reactivation. We also noticed a spatial hierarchy in the immune infiltrates, with high levels of CD137+CD8+ T cells producing IFNγ and CD137+M1 cells producing IL12 in the tuberculoma. These findings indicate that changes in the tumor’s stromal tissue, linked to ICI responsiveness, can impact the composition and distribution of immune infiltrates. In circumstances where this balance is overthrown, a specialized stromal niche shift occurs, bringing CD137+ IFNγ+ CD8+ T cells and CD137+ IL12+ M1 macrophages closer together, increasing the likelihood of TB reactivation (Figure 6D). Overall, our study highlights the risks associated with checkpoint blockade in cancer patients and suggests that the development of CD137 agonists for clinical use should consider these issues.

Polyfunctional T cells have been associated with both protection and disease activity in TB studies [37,38,39,40,41]. While TNFα can accelerate MTB growth [32], higher levels of IFNγ were found in the lungs of MTB-infected PD-1 KO mice compared to wild-type mice [42]. Our study confirms the IFNγ findings and further reveals the enrichment of IFNγ-producing CD8+ T cells in tuberculoma tissues and their correlation with IL12-producing M1 cells. This is the first time that cytokine production from innate cells has been shown to be correlated with adaptive cells in PD1-related TB tissues. We suggest that further studies consider more comprehensive innate immune profiles within anti-PD1-related granuloma whenever possible. 

CD137+ T cells present at the tumor site have been found to be associated with the strength of the anti-tumor immune response mainly expressed by tumor-infiltrating lymphocytes that release IFNγ [43]. Also, the co-expression of CD137 and PD-1 influences the activation of T cells [44]. Public datasets have shown that high levels of CD137hiIFNγhi CD8+ T cells are linked to a high response rate, suggesting CD137 may be a potential biomarker for predicting the effectiveness of cancer immunotherapy [44,45,46]. We observed intense CD137 expression on IFNγ-producing CD8+ T cells in the PD-1-induced tuberculoma, suggesting the role of the CD137 axis in regulating the adaptive immune response during TB infection, aligning with previous research [47]. Also, we propose the modulation of CD137 expression on CD8+ T cells may be influenced by PD-1-mediated signaling rather than specific TB antigens or inflammatory cytokines (Figure S7A,B and supplementary information). However, it is still unknown whether these IFNγ+ CD137+ CD8+ T cells are specific to TB antigens or non-specific bystander T cells in the tuberculoma. Moreover, it is important to note that our k-means clustering analysis successfully grouped tissue samples into subgroups, and interestingly, clusters 1–3 included samples both from younger (in their 20s) and older (in their 60s) subjects. This finding suggests that the TSIME plays a significant role in determining immune response in tuberculoma, potentially overriding age-related differences in immune cell compartments and functionality. Therefore, it is crucial to consider the unique immune landscapes of specific tissues when studying immune response in tuberculoma. However, it is worth noting that aging is a significant risk factor for TB, primarily due to waning immunity in the elderly [48,49]. Consequently, age-related differences in immune responses cannot be ignored in the context of TB, and future studies should explore the extent to which these differences contribute in shaping immune response in tuberculoma

The immune contexture has been introduced to describe how different elements in the TME are spatially arranged and predicts the clinical outcome of cancers. Our study found that CD137hiIFNγhi CD8+ T cells were close to CD137hiIL12hi M1 cells, and this cell interaction was maintained in the TME of ICI-responsive NSCLC patients but not in non-ICI-related TB disease. Cells within a distance range of 0–50 µm were more likely to interact [50], suggesting a zone in ICI-associated tuberculoma for CD8+ T cells to undergo functional changes in situ. We believe that inhibiting PD-1-signaling could activate CD137 expression, creating a reservoir of immune cells with inflammatory functions and facilitating TB reactivation. If CD137-rich immune subsets “shift” towards one that supports mycobacteria growth, away from a cancer-supporting niche, TB reactivation may be triggered. Further research is needed to understand the phenotypic plasticity of the CD137+ population, their role in tumor growth or destruction, their distribution across different tissue types, and their role within the granuloma. Moreover, our findings strongly suggest that histopathological evaluation of CD137 in various pathological tissue lesions, regardless of anatomical distribution or tissue origins, can be a critical method for oncologists to predict tumor response assessment and effectively monitor the response to immunologic treatments. This knowledge also provides a partial explanation for the heterogeneous responses observed clinically, which were inadequately documented by the limited tomographic measurements provided by the Response Evaluation Criteria in Solid Tumors (RECIST).

Our study has several limitations despite the promising results of our small proof-of-concept profiling experiment. The analysis was restricted by the limited number of specimens and the selection of biopsies from different time-points in only two NPC patients. Obtaining tissue biopsies from cancer patients undergoing checkpoint blockade treatment and also having TB posed significant challenges for comprehensive research in this specific patient population. Furthermore, the scarcity of non-cancer-related tuberculoma samples limited our exploration of the immunological aspects of anti-PD-1-related tuberculoma in NPC patients. To overcome this limitation, we analyzed public whole blood transcriptomic datasets from patients with active TB. Additionally, we faced difficulties in collecting control samples from cancer patients who showed a favorable tumor response without developing TB. Therefore, we compared ICI response rates across different cancer types to understand immune phenotypic heterogeneity and spatial distribution patterns in the tumor microenvironment of cancer patients without TB, and compared these findings with our TB-associated cancer cohort. Retrospective studies have indicated that cancer patients who developed TB during ICI treatment had more positive responses [8]. Future studies could analyze patients who had latent TB at the time of ICI treatment but did not respond to gain a deeper understanding of the mechanisms underlying TB reactivation in response to ICI-induced changes in the tumor microenvironment. 

## 5. Conclusions

Despite the limited number of patients in this study, we provide proof-of-concept that the absence of CD137 biosignatures on tumor-infiltrating CD8+ T cells in response to PD-1 inhibitors may predict TB reactivation. While the underlying mechanism remains unknown, these inflammatory responses at different pathological lesions preserve distinct TSIMEs that can influence the response to therapy within their own niche. The observed CD137-niche shift in the PD-1-driven tuberculoma highlights the significance of tissue-dependent difference in therapeutic responses. However, this hypothesis requires further investigation in a larger cohort of patients for validation.

## Data Availability

All data are available in the main text or the supplementary material.

## Supplementary Materials

The following supporting information can be downloaded at: https://www.mdpi.com/article/10.3390/cancers16020268/s1. Figure S1: Multiplex IHC quality control and validation. Figure S2: Multiplex IHC to visualize cell density. Figure S3: Characteristics of the two non-tumor tissue outliers. Figure S4: Multiplex IHC to visualize T cell activation. Figure S5: Heterogeneous structured immune microenvironments across two different non-tumor tissues. Figure S6: Cellular interconnectivity in granuloma tissue. Figure S7: Generating three-dimensional human granulomas in vitro to study cytokine in tuberculosis. Table S1: Antibody List. Table S2: Lineage and Identification Table. Table S3: Staining Sequence for Oncogenic Panel. Table S4: Sample Information and Corresponding Grouping. Table S5: Staining Sequence for Myeloid Panel. Table S6: Staining Sequence for Lymphoid Panel. Table S7: Staining Sequence for Functional Panel. Table S8: Staining Sequence for Molecular Panel. Table S9: Bubble Plot Data for NT-P (Outlier 1). Table S10: Bubble Plot Data for NT-I (Outlier 2). Table S11: Bubble Plot Data for Cluster 1 (NT). Table S12: Bubble Plot Data for Cluster 2 (M). Table S13: Bubble Plot Data for Cluster 3 (G). Table S14: Mean Range Table for Cluster 1 (NT); Cluster 2 (M); Cluster 3 (G). Table S15: Mean Range Table Outlier 1 (NT-P), Outlier 2 (NT-I). Table S16: R Package.
