# Supplementary material for: Shift in Tissue-Specific Immune Niches and CD137 Expression in Tuberculoma of Pembrolizumab-Treated Nasopharyngeal Carcinoma Patients
Source: Cancers (Basel). 2024 Jan 8;16(2):268. doi: 10.3390/cancers16020268 (PMC10813936; doi:10.3390/cancers16020268)
Supplement: Supplementary file 1 [file cancers-16-00268-s001.zip › cancers-2787072-supplementary.pdf]

SUPPLEMENTARY FIGURES

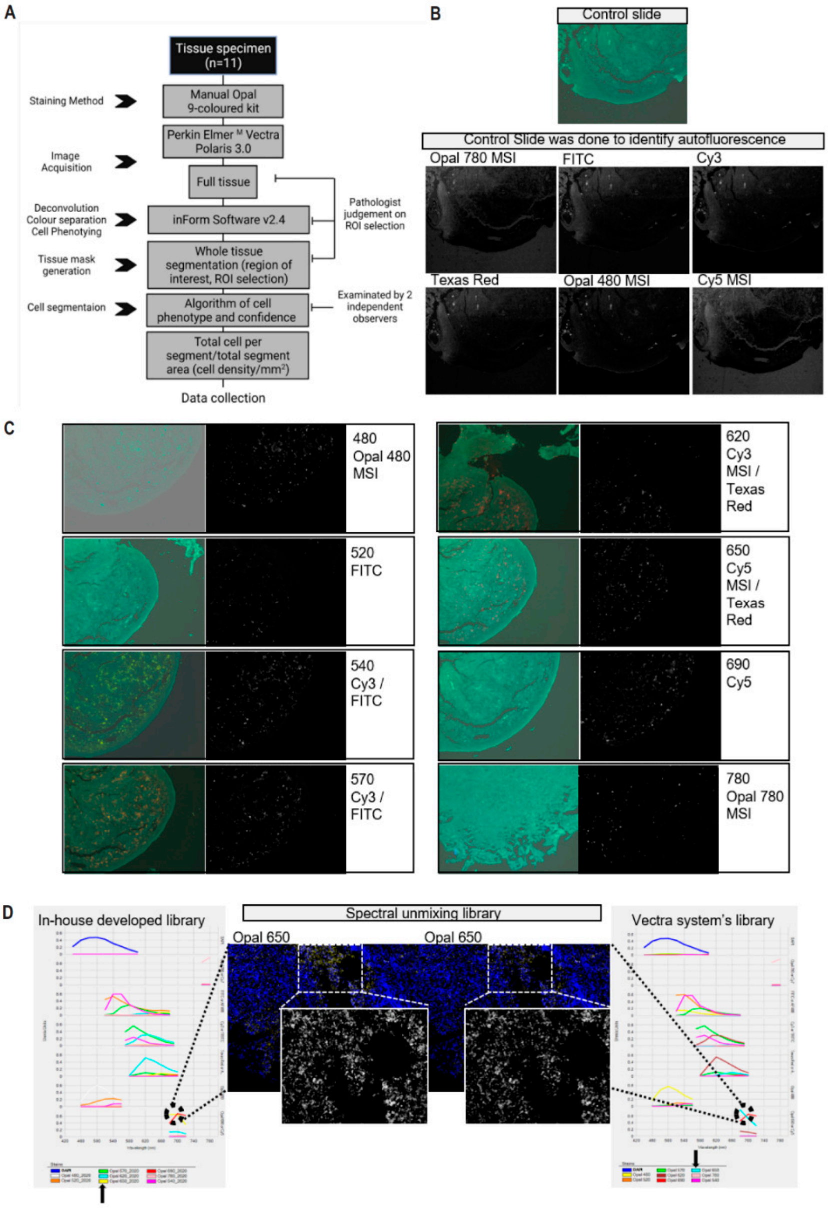

Supplementary Figure S1. Multiplex IHC quality control and validation.**(A)** Multiplex IHC experimental overview and example of pipeline for quantification of immune subsets in tissue biopsies. **(B)** The images display the removed autofluorescence for acquiring multispectral (MSI) regions of interest in control slide. **(C)** Raw acquired images from for spectral decomposition ("unmixing") with corresponding Opal™ dyes in control slide. **(D)** Example of in-house developed library yields very accurate unmixing results. Fluorescence image of NPC control tissue stained against PD-L1 with Opal 650 dye with Vectra systems' library (right). Emission spectra of purer Opal 650 (yellow line: in-house library) compared to the less pure Opal 650 (turquoise: Vectra's library). The established in-house spectral library was used for acquisition and analysis of future datasets.

A

B

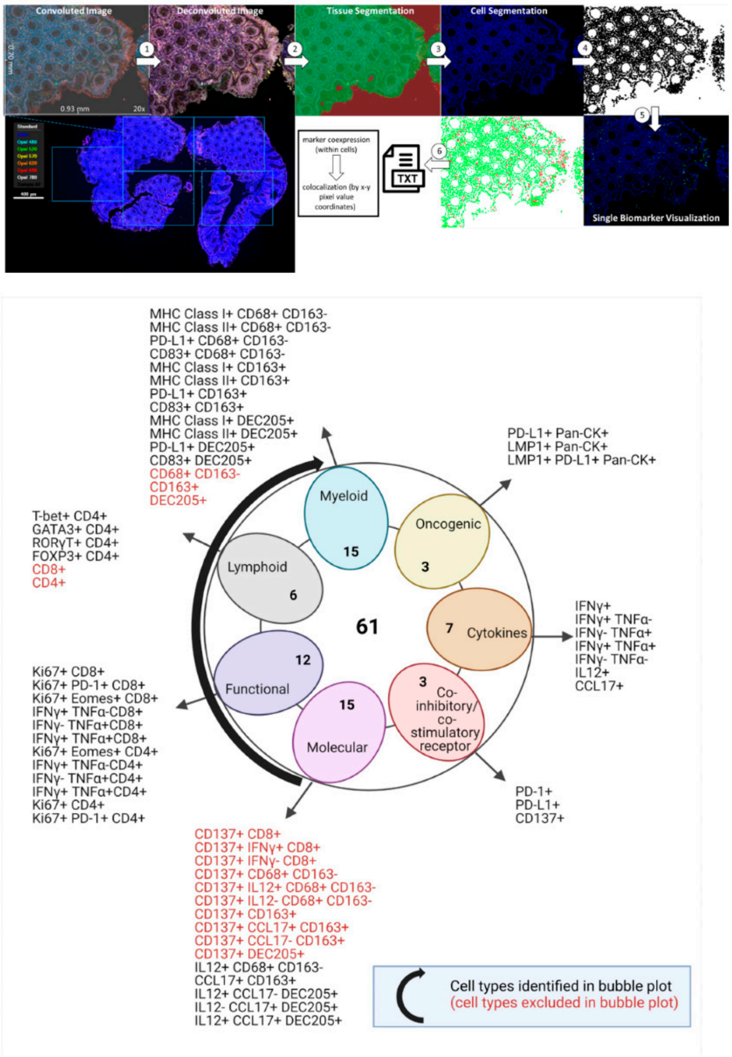

Supplementary Figure S2. Multiplex IHC to visualize cell density. **(A)** Workflow of experimental multiplex IHC study. **(B)** 61-biomarker combinations [48 combinations identified for immune subsets (identified from myeloid, lymphoid, functional and molecular panels), 3 combinations identified for cancer types (identified from oncogenic panel); 7 combination identified for cytokine patterns and 3 identified for co-inhibitory/co-stimulatory receptor)] were used to map relatively cell composition and states across clinical specimen. Black arrow represented 33 immune cell types applied in the bubble plots of Fig. 6B. Red highlighted cell types were excluded from the bubble plots (including 5 pan-immune cell markers: $CD4^{+}$, $CD8^{+}$, $CD68^{+}CD163^{-}$, $CD163^{+}$ and $DEC205^{+}$, which were chosen to exclude due to high proportion these major classes of immune cells compared to the remaining anaylzed cells; and 10 CD137-expressing cells, which were used for the refinement panels)

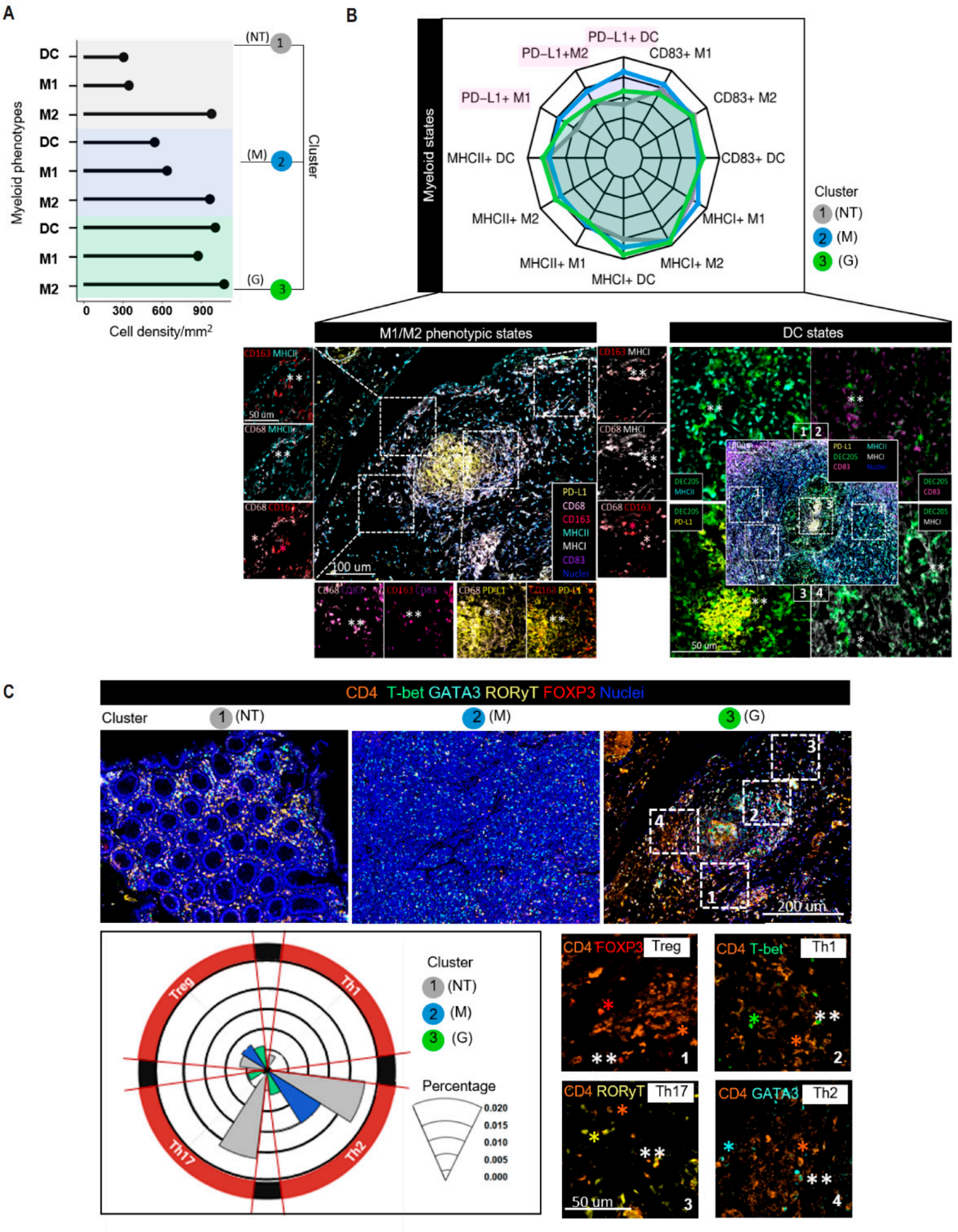

Supplementary Figure S3. The phenotype and activation status of myeloid immune cells. **(A)** Myeloid cell densities (line plot; dendritic cells, DC; type 1 macrophages, M1; type 2 macrophages, M2). **(B)** Myeloid cell in activation status (spider plot; PDL1, MHCI, MHCII). Down-regulated cell types associated with cluster 3 (G) highlighted in red. Representative images of co-expressed markers (DEC205/MHCI, DEC205/MHCII, DEC205/PD-L1, DEC205/CD83) and colocalized markers of MHCI/MHCII/CD68/PDL1 with CD68/CD163. Colocalized markers $CD68^{+}CD163^{-}$ were indicated as M1, and $CD68^{-}CD163^{+}$ as M2. Zoom in is indicated by white squares. Exemplary cells are annotated with respective color-coded asterisks, where (∗) indicated as single-positive marker and (∗∗) identified as double-positive biomarkers. **(C)** Representative image from cluster 1 (NT), cluster 2 (M) and cluster 3 (G). Biomarkers and their corresponding colors shown. Boxes in dotted lines represent the selected regions of cluster 3. Nightingale rose plot showed the median frequency of helper 1 T- (Th1; $CD4^{+}Tbet^{+}$), helper 2 T- (Th2; $CD4^{+}GATA^{+}$), helper 17 T- (Th17; $CD4^{+}ROR\gamma t^{+}$) and regulatory T- (Treg; $CD4^{+}Foxp3^{+}$) cells. The arcs around the circumference denote CD4 sub-populations. Exemplary cells were annotated with the respective color-coded, where (∗) indicated as single-positive marker and (∗∗) identified as double-positive biomarkers

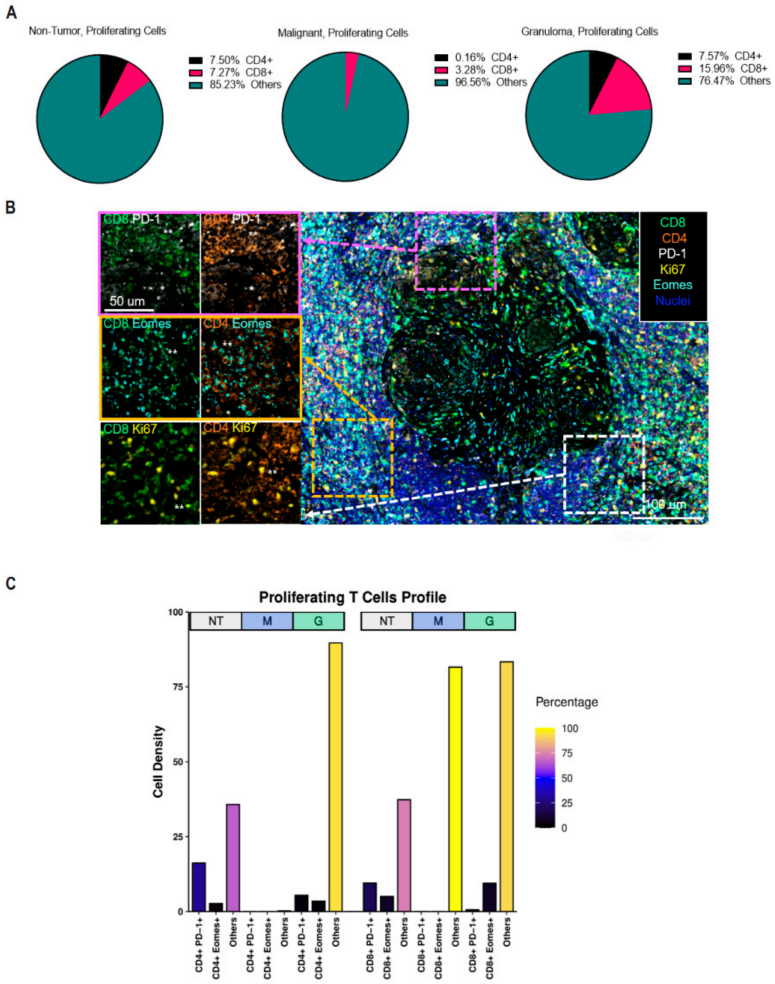

Supplementary Figure S4. Multiplex IHC to visualize T cell activation. **(A)** Pie chart showing the proportion of proliferating CD4⁺ and CD8⁺ T cells in non-tumor, tumor and granuloma tissues. **(B)** Representative images from granulomatous tissue. Biomarkers and colors are shown. Colored boxes show the magnified area. **(C)** Bar plot showing the cell density (cell density/mm^2) of different T-cell subsets in cluster 1 (NT), cluster 2 (M) and cluster 3 (G) tissue types. The color gradient represents the percentage of each T cell subpopulations within total CD4$^+$ T cells or total CD8$^+$ T cells.

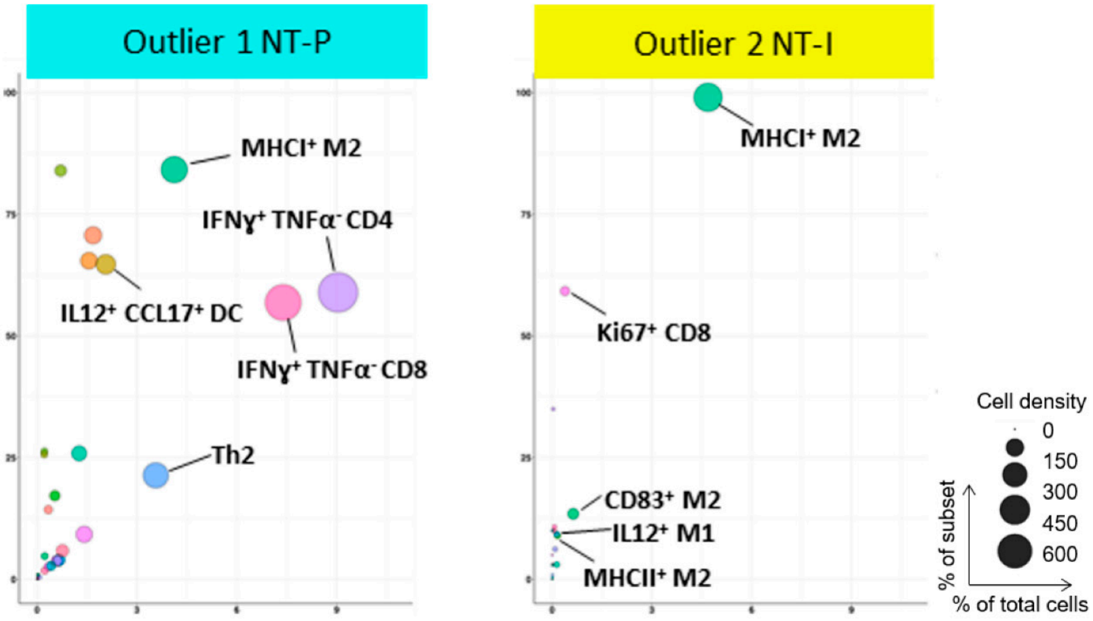

Supplementary Figure S5. Heterogeneous structured immune microenvironments across two different non-tumor tissues. Bubble-chart plots summarized frequencies of all cell types in outlier 1 (NT-P) and outlier 2 (NT-I). The bubble size was proportional to the cell density. X-axis represents the percentage of indicated cellular subset in total cells, and y-axis indicates the cellular subset in myeloid-/lymphoid linage. All the different cellular subsets are color coded.

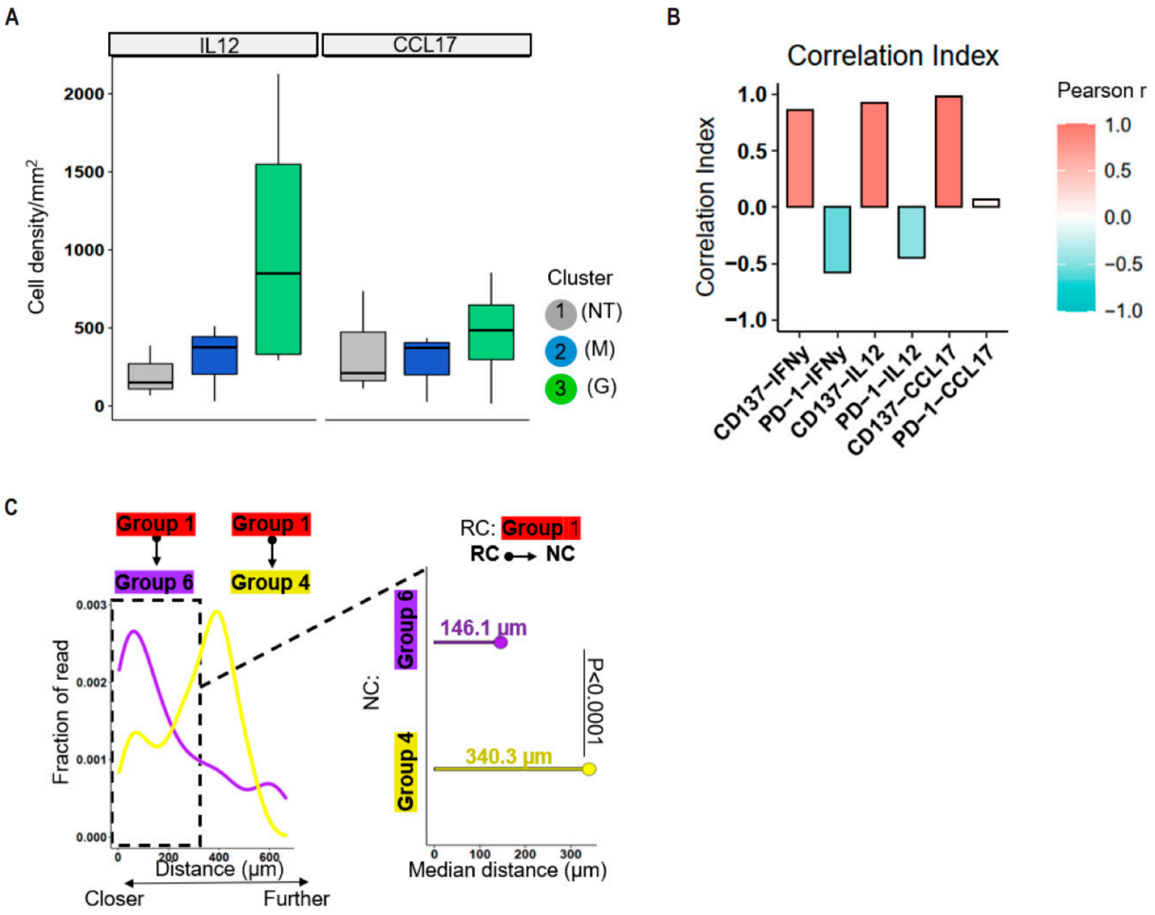

Supplementary Figure S6. Cellular interconnectivity in granuloma tissue. **(A)** Boxplots displaying the total cell density of IL12 and CCL17 and PD-1 expression in cluster 1 (NT), cluster 2 (M), cluster 3 (G) tissue types. **(B)** Pearson correlation for CD137, PD1, IFNγ, IL12 and CCL17 for all cells across within granuloma tissue. Color indicates direction of change, that is, red=gained association, green=lost association. **(C)** Histograms show distances in µm Histograms show all (left) or given distance (right) in µm of between reference cell (RC) type group and nearest cell (NC) type group within granuloma. Solid dot represents RC (Group 1 cell type in green: CD137+IFNγ-CD8) and arrow line represent NC (Group 4 cell types in purple: CD137+IL12+M1 or group 6 cell types in yellow: CD137+IL12+M2). The dashed rectangle highlights the most differential distribution between respective RC to NC. Data information: In the boxplots in (A), the centerline indicates the median, while the upper and lower lines represent the 75th and 25th percentiles, respectively. In (C), median value of the nearest distances (range from 0 to 300 µm) indicated in the plot. P values were calculated with a Wilcoxon test.

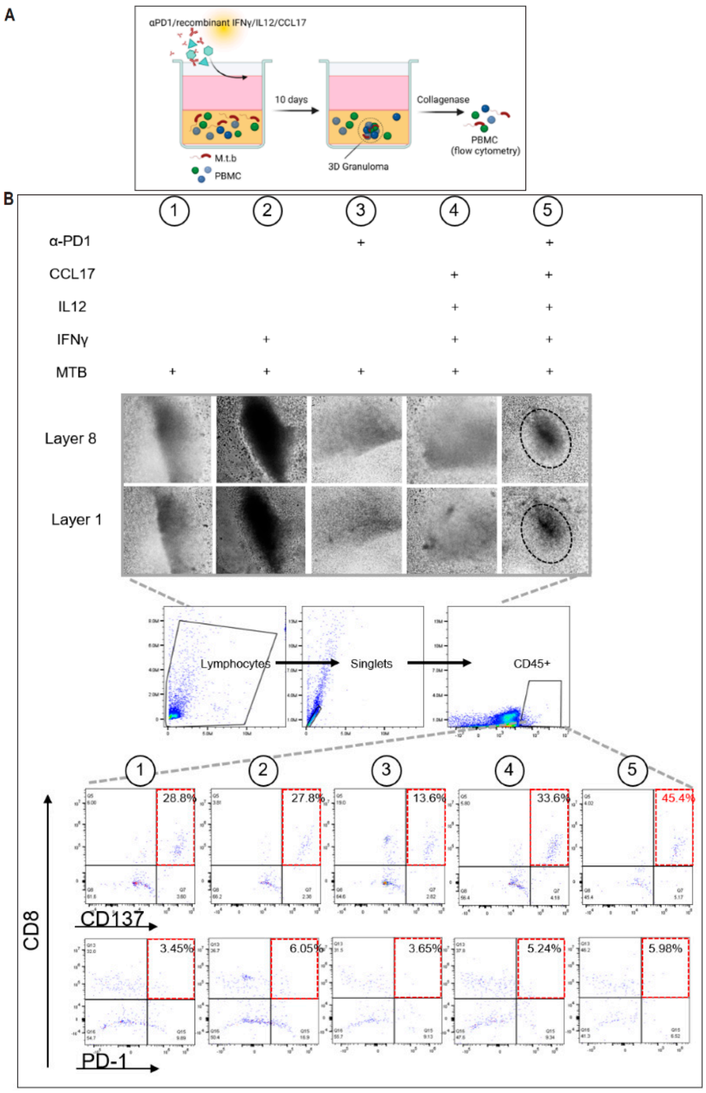

Supplementary Figure S7. Generating three-dimensional human granulomas *in vitro* to study cytokine in tuberculosis.**(A)** Schematic representation of the 3D *in vitro* granuloma model. Five different treatment conditions in the presence or absence of recombinant MTB antigens ESAT-6/CFP-10, recombinant cytokine proteins (IFNγ/IL12/CCL17) or KEYTRUDA® (pembrolizumab, anti-PD1 inhibitor; αPD1) are cultured with PBMCs and embedded in an extracellular matrix. Formation of granuloma-like structures can be observed after 10 days. PBMCs can be released from the extracellular matrix by collagenase digestion for subsequent flow cytometric analysis. (**B**) *In vitro* granuloma-like structures are formed on day 10 by the combination of different treatment (numbered), including the addition of recombinant MTB antigens and/or recombinant IFNγ and/or recombinant IL12 and/or recombinant CCL17 and/or anti-PD1 inhibitor (indicated as "+" and "–" in the legends). Images of the granulomas at day 10 revealed multilayered structures containing approximately 8 cell layers. No formation of granulomas was observed in all other treatment conditions. Gating strategy to identify the principle CD45^{+} immune cells. CD45 gated for CD8 T cell. CD8 T cells were then separated into two subsets (CD137^{+} CD8 T cells and PD1^{+} CD8 T cells) based on CD137 and PD1 labeling.

SUPPLEMENTARY TABLES

Supplementary Table S1 Antibody List

**Antibody Name**	**Company**	**Reference Number**
CCL17	Abcam	Ab182793
CD4	DAKO	M7310
CD8	DAKO	IS623
CD68	DAKO	M0814
CD83	ThermoFisher Scientific	PA5-80448
CD137	Invitrogen	MA5-13739
CD163	ThermoFisher Scientific	MS-1103-S0
DEC-205	LSBio	B2669
Eomes	Millipore	AB2283
FOXP3	Abcam	Ab20034
GATA3	BD Pharmingen	558686
IFN	Abcam	Ab9657
IL12	Abcam	Ab131039
Ki67	Abcam	Ab15580
LMP1	Enzo	ALX-810-231-R500
MHC Class I	Abcam	Ab70328
MHC Class II	Novus	SPM288
MTB	Abcam	Ab905
Pan-CK	DAKO	M0821
PD-1	Abcam	Ab52587
PD-L1	DAKO	M3653
ROR	Milipore	MABF81
T-bet	Abcam	Ab91109
TNF	Abcam	Ab1793

Supplementary Table S2 Lineage and Identification Table

**T helper cell**	
CD4$^+$	T helper cells
Ki67$^+$ CD4$^+$	Proliferating T helper cells
Ki67$^+$ PD-1$^+$ CD4$^+$	Proliferating Suppressive T helper cells
T-bet$^+$ CD4$^+$	Th1
GATA3$^+$ CD4$^+$	Th2
ROR T$^+$ CD4$^+$	Th17
FOXP3$^+$ CD4$^+$	Treg
Ki67$^+$ Eomes$^+$ CD4$^+$	Proliferating Eomes expressing T helper cells
IFN $^+$ TNF CD4$^+$	IFN releasing T helper cells
IFN $^-$ TNF CD4$^+$	TNF releasing cells
IFN $^+$ TNF CD4$^+$	IFN and TNF releasing T helper cells
**CD8 positive T cells**	
CD8$^+$	CD8 positive T cells
Ki67$^+$ CD8$^+$	Proliferating CD8 positive T cells
Ki67$^+$ PD-1$^+$ CD8$^+$	Proliferating Suppressive CD8 positive T cells
Ki67$^+$ Eomes$^+$ CD8$^+$	Proliferating CD8 positive memory T cells
IFN $^+$ TNF CD8$^+$	IFN releasing CD8 positive T cells (non-TNF releasing)
IFN $^-$ TNF CD8$^+$	TNF releasing CD8 positive T cells (non- IFN releasing)
IFN $^+$ TNF CD8$^+$	IFN and TNF releasing CD8 positive T cells
CD137$^+$ CD8$^+$	CD137 positive CD8 positive T cells
CD137$^+$ IFN $^+$ CD8$^+$	CD137 positive IFN releasing CD8 positive T cells
CD137$^+$ IFN $^-$ CD8$^+$	CD137 positive non-IFN releasing CD8 positive T cells
**Macrophage-M1**	
CD68$^+$ CD163$^-$	M1 cells
MHC Class I$^+$ CD68$^+$ CD163$^-$	Antigen presenting M1 cells
MHC Class II$^+$ CD68$^+$ CD163$^-$	Antigen presenting M1 cells
PD-L1$^+$ CD68$^+$ CD163$^-$	Immune suppressive M1 cells
CD83$^+$ CD68$^+$ CD163$^-$	Activated M1 cells
IL12$^+$ CD68$^+$ CD163$^-$	IL12 releasing M1 cells
CD137$^+$ CD68$^+$ CD163$^-$	CD137 positive M1 cells
CD137$^+$ IL12$^+$ CD68$^+$ CD163$^-$	CD137 positive IL12 releasing M1 cells
CD137$^+$ IL12$^-$ CD68$^+$ CD163$^-$	CD137 positive non-IL12 releasing M1 cells
**Macrophage-M2**	
CD163$^+$	M2 cells
MHC Class I$^+$ CD163$^+$	Antigen presenting M2 cells
MHC Class II$^+$ CD163$^+$	Antigen presenting M2 cells
PD-L1$^+$ CD163$^+$	Suppressive M2 cells
CD83$^+$ CD163$^+$	Activated M2 cells
CCL17$^+$ CD163$^+$	CCL17 releasing M2 cells
CD137$^+$ CD163$^+$	CD137 positive M2 cells
CD137$^+$ CCL17$^+$ CD163$^+$	CD137 positive CCL17 releasing M2 cells
CD137$^+$ CCL17$^-$ CD163$^+$	CD137 positive non-CCL17 releasing M2 cells
**Dendritic cells**	
DEC205$^+$	Dendritic cells
MHC Class I$^+$ DEC205$^+$	Antigen presenting dendritic cells

MHC Class II^{+} DEC205^{+}	Antigen presenting dendritic cells
PD-L1^{+} DEC205^{+}	Immune suppressive dendritic cells
CD83^{+} DEC205^{+}	Activated dendritic cells
IL12^{+} CCL17^{-} DEC205^{+}	IL12 releasing dendritic cells
IL12^{-} CCL17^{+} DEC205^{+}	CCL17 releasing dendritic cells
IL12^{+} CCL17^{+} DEC205^{+}	IL12 and CCL17 releasing dendritic cells
CD137^{+} DEC205^{+}	CD137 positive dendritic cells
**Cancer cells**	
PD-L1^{+} Pan-CK^{+}	Immune suppressive cancer cells
LMP1^{+} Pan-CK^{+}	EBV infected cancer cells
LMP1^{+} PD-L1^{+} Pan-CK^{+}	Immune suppressive EBV infected cancer cells
**Cytokines**	
IFN $^{+}$	IFN releasing cells
IFN $^{+}$ TNF	IFN releasing cells (non-TNF releasing)
IFN $^{-}$ TNF	TNF releasing cells (non- IFN releasing)
IFN $^{+}$ TNF	IFN and TNF releasing cells
IFN $^{-}$ TNF	Non-IFN and non-TNF releasing cells
IL12^{+}	IL1 releasing cells
CCL17^{+}	CCL17 releasing cells
**Co-inhibitory/co-stimulatory receptor**	
PD-1^{+}	PD-1 positive cells
PD-L1^{+}	PD-L1 positive cells
CD137^{+}	CD137 positive cells

Supplementary Table S3 Staining Sequence for Oncogenic Panel

Antibody	Dilution	Protein Block Time	Antigen Retrieval Buffer	Opal Dye	Incubation Time
LMP1	1:100	10 minutes	Citric Acid	Opal 650	4ºC Overnight
PD-L1	1:50	10 minutes	Citric Acid	Opal 690	Room Temperature for 1 hour
PD-1	1:50	10 minutes	Citric Acid	Opal 480	4ºC Overnight
IFN	1:1000	10 minutes	Citric Acid	Opal 620	4ºC Overnight
Pan-CK	1:100	10 minutes	Citric Acid	Opal 540	Room Temperature for 1 hour
Number of biomarkers: 5					
Number of combinations: 6					
Cell types: [3] PD-L1$^+$ Pan-CK$^+$, LMP1$^+$ Pan-CK$^+$, LMP1$^+$ PD-L1$^+$ Pan-CK$^+$					
Cytokine releasing cells: [1] IFN $^+$					
Co-inhibitory/co-stimulatory receptor: [2] PD-1$^+$, PD-L1$^+$					

Supplementary Table S4 Sample Information and Corresponding Grouping

Patient	Lab Biopsy Number	Biopsy Description	Sample Grouping
I	674	Initial NPC biopsy	Cluster 2 (M)
I	824	Ileum biopsy with granuloma inflammation	Cluster 3(G)
I	307	Nasopharyngeal biopsy before resuming pembrolizumab after completion of anti-TB medication.	Outlier 2 (NT-I)
I	309	Nasopharyngeal biopsy after resuming pembrolizumab after completion of anti-TB medication.	Cluster 1 (NT)
I	296A	Terminal ileum biopsy 9 months after the completion of anti-TB medication	Cluster 1 (NT)
I	296B	Terminal cecum biopsy 9 months after the completion of anti-TB medication	Cluster 1 (NT)
II	208	Nasopharyngeal baseline sample	Outlier 1 (NT-P)
II	174	Lung biopsy with undifferentiated NPC	Cluster 2 (M)
II	629A	Liver biopsy with undifferentiated NPC and granuloma inflammation	Cluster 2 (M) and Cluster 3 (G)
II	629B	Falciform ligament biopsy with granuloma inflammation	Cluster 3 (G)
II	629C	Duodenal lymph node with granuloma inflammations	Cluster 3 (G)

Supplementary Table S5 Staining Sequence for Myeloid Panel

Antibody	Dilution	Protein Block Time	Antigen Retrieval Buffer	Opal Dye	Incubation Time
DEC-205	1:150	10 minutes	Citric Acid	Opal 570	4ºC Overnight
PD-L1	1:50	10 minutes	Citric Acid	Opal 650	Room Temperature for 1 hour
MHC Class I	1:100	10 minutes	Tris-EDTA	Opal 480	Room Temperature for 1 hour
CD163	1:50	30 minutes	Citric Acid	Opal 690	4ºC Overnight
MHC Class II	1:400	30 minutes	Citric Acid	Opal 620	4ºC Overnight
CD83	1:1000	30 minutes	Citric Acid	Opal 540	Room Temperature for 1 hour
CD68	1:100	30 minutes	Citric Acid	Opal 780	4ºC Overnight
Number of biomarkers: 7					
Number of combinations: 15					
Cell types: [15] $DEC205^{+}$, MHC Class I^{+} $DEC205^{+}$, MHC Class II^{+} $DEC205^{+}$, PD-$L1^{+}$ $DEC205^{+}$, $CD83^{+}$ $DEC205^{+}$, $CD68^{+}$ $CD163^{-}$, MHC Class I^{+} $CD68^{+}$ $CD163^{-}$, MHC Class II^{+} $CD68^{+}$ $CD163^{-}$, PD-$L1^{+}$ $CD68^{+}$ $CD163^{-}$, $CD83^{+}$ $CD68^{+}$ $CD163^{-}$, $CD163^{+}$, MHC Class I^{+} $CD163^{+}$, MHC Class II^{+} $CD163^{+}$, PD-$L1^{+}$ $CD163^{+}$, $CD83^{+}$ $CD163^{+}$					

Supplementary Table S6 Staining Sequence for Lymphoid Panel

Antibody	Dilution	Protein Block Time	Antigen Retrieval Buffer	Opal Dye	Incubation Time
CD4	1:50	1 hour	Tris-EDTA	Opal 520	4ºC Overnight
CD8	NA	10 minutes	Tris-EDTA	Opal 570	Room Temperature for 1 hour
ROR	1:200	10 minutes	Tris-EDTA	Opal 650	Room Temperature for 1 hour
GATA3	1:800	10 minutes	Tris-EDTA	Opal 690	Room Temperature for 1 hour
FOXP3	1:100	10 minutes	Tris-EDTA	Opal 620	4ºC Overnight
T-bet	1:100	10 minutes	Citric-Acid	Opal 540	Room Temperature for 1 hour
Number of biomarkers: 6					
Number of combinations: 6					
Cell types: [6] $CD4^+$, $T\text{-}bet^+$ $CD4^+$ (Th1), $GATA3^+$ $CD4^+$ (Th2), ROR T^+ $CD4^+$ (Th17), $FOXP3^+$ $CD4^+$ (Treg), $CD8^+$					

Supplementary Table S7 Staining Sequence for Functional Panel

Antibody	Dilution	Protein Block Time	Antigen Retrieval Buffer	Opal Dye	Incubation Time
CD4	1:50	1 hour	Tris-EDTA	Opal 520	4ºC Overnight
CD8	NA	10 minutes	Tris-EDTA	Opal 570	Room Temperature for 1 hour
Ki-67	1:200	10 minutes	Citric Acid	Opal 650	Room Temperature for 1 hour
PD-1	1:50	10 minutes	Citric Acid	Opal 480	4ºC Overnight
Eomes	1:300	10 minutes	Citric Acid	Opal 620	Room Temperature for 1 hour
IFN	1:1000	10 minutes	Citric Acid	Opal 690	4ºC Overnight
T-bet	1:100	10 minutes	Citric Acid	Opal 540	Room Temperature for 1 hour
TNF	1:100	10 minutes	Citric Acid	Opal 780	4ºC Overnight
Number of biomarkers: 8					
Number of combinations: 19					
Cell types: [14] $CD4^+$, $Ki67^+$ $CD4^+$, $Ki67^+$ $PD\text{-}1^+$ $CD4^+$, $Ki67^+$ $Eomes^+$ $CD4^+$, IFN^+ TNF $CD4^+$, IFN^- TNF $CD4^+$, IFN^+ TNF $CD4^+$, $CD8^+$, $Ki67^+$ $CD8^+$, $Ki67^+$ $PD\text{-}1^+$ $CD8^+$, $Ki67^+$ $Eomes^+$ $CD8^+$, IFN^+ TNF $CD8^+$, IFN^- TNF $CD8^+$, IFN^+ TNF $CD8^+$					
Cytokine releasing cells: [4] IFN^+ TNF IFN^- TNF IFN^+ TNF IFN^- TNF					
Co-inhibitory/co-stimulatory receptor: [1] $PD\text{-}1^+$					

Supplementary Table S8 Staining Sequence for Molecular Panel

Antibody	Dilution	Protein Block Time	Antigen Retrieval Buffer	Opal Dye	Incubation Time
CD137	1:50	10 minutes	Citric Acid	Opal 620	4ºC Overnight
CD8	NA	10 minutes	Tris-EDTA	Opal 520	Room Temperature for 1 hour
DEC205	1:150	10 minutes	Citric Acid	Opal 570	4ºC Overnight
IL12	1:250	10 minutes	Citric Acid	Opal 540	Room Temperature for 1 hour
CD163	1:50	30 minutes	Citric Acid	Opal 650	4ºC Overnight
IFN	1:1000	10 minutes	Citric Acid	Opal 690	Room Temperature for 1 hour
CCL17	1:50	30 minutes	Citric Acid	Opal 480	4ºC Overnight
CD68	1:100	30 minutes	Citric Acid	Opal 780	4ºC Overnight
Number of biomarkers: 8					
Number of combinations: 18					
Cell types: [15] IL12^{+} CCL17^{-} DEC205^{+}, IL12^{-} CCL17^{+} DEC205^{+}, IL12^{+} CCL17^{+} DEC205^{+}, CD137^{+} DEC205^{+}, IL12^{+} CD68^{+} CD163^{-}, CD137^{+} CD68^{+} CD163^{-}, CD137^{+} IL12^{-} CD68^{+} CD163^{-}, CD137^{+} IL12^{+} CD68^{+} CD163^{-}, CCL17^{+} CD163^{+}, CD137^{+} CD163^{+}, CD137^{+} CCL17^{-} CD163^{+},CD137^{+} CCL17^{+} CD163^{+}, CD137^{+} CD8^{+}, CD137^{+} IFN $^{-}$ CD8^{+},CD137^{+} IFN $^{+}$ CD8^{+}					
Cytokine releasing cells: [2] IL12^{+}, CCL17					
Co-inhibitory/co-stimulatory receptor: [1] CD137^{+}					

Supplementary Table S9 Bubble Plot Data for NT-P (Outlier 1)

**Cell Type**	**Percentage Out of Total Cell**	**Percentage Out of Subcategory**	**Cell Density**
IFN $^{+}$ TNF $^{-}$ CD4	9.042%	58.952%	1497.442
IFN $^{+}$ TNF $^{-}$ CD8	7.395%	56.874%	1224.578
MHC Class I^{+} M2	4.122%	84.181%	651.595
Th2	3.569%	21.358%	591.295
IL12^{+} CCL17^{+} DC	2.066%	64.717%	349.560
MHC Class I^{+} DC	1.685%	70.682%	266.289
MHC Class II^{+} DC	1.560%	65.455%	246.596
IFN $^{+}$ TNF $^{+}$ CD4	1.418%	9.245%	234.828
MHC Class II^{+} M2	1.267%	25.885%	200.359
IFN $^{+}$ TNF $^{+}$ CD8	0.764%	5.876%	126.510
MHC Class I^{+} M1	0.710%	83.974%	112.167
Th17	0.669%	4.001%	110.770
Treg	0.636%	3.804%	105.310
IFN $^{-}$ TNF $^{+}$ CD4	0.584%	3.809%	96.743
IL12^{+} M1	0.536%	17.181%	90.702
Ki67^{+} CD4	0.424%	2.77%	70.283
CD83^{+} DC	0.341%	14.318%	53.943
Ki67^{+} CD8	0.339%	2.611%	56.226
CD83^{+} M2	0.233%	4.757%	36.818
IFN $^{-}$ TNF $^{+}$ CD8	0.230%	1.767%	38.036
MHC Class II^{+} M1	0.222%	26.282%	35.106
CD83^{+} M1	0.217%	25.641%	34.249
Th1	0.075%	0.451%	12.481
IL12^{-} CCL17^{+} DC	0.030%	0.943%	5.096
CCL17^{+} M2	0.030%	0.876%	5.096
Ki67^{+} Eomes^{+} CD8	0.020%	0.154%	3.307
Ki67^{+} Eomes^{+} CD4	0.010%	0.065%	1.654
IL12^{+} CCL17^{-} DC	0.000%	0.000%	0
PD-L1^{+} M1	0.000%	0.000%	0
PD-L1^{+} M2	0.000%	0.000%	0

PD-L1$^+$ DC	0.000%	0.000%	0
Ki67$^+$ PD-1$^+$ CD4	0.000%	0.000%	0
Ki67$^+$ PD-1$^+$ CD8	0.000%	0.000%	0

Supplementary Table S10 Bubble Plot Data for NT-I (Outlier 2)

Cell Type	Percentage Out of Total Cell	Percentage Out of Subcategory	Cell Density
MHC Class I^{+} M2	4.688%	99.030%	754.936
CD83^{+} M2	0.637%	13.455%	102.568
Ki67^{+} CD8	0.389%	59.230%	65.823
IL12^{+} M1	0.164%	8.984%	29.827
MHC Class II^{+} M2	0.143%	3.030%	23.101
Treg	0.139%	9.292%	22.487
Th1	0.093%	6.195%	14.991
IFN $^{+}$ TNF $^{-}$ CD8	0.071%	10.769%	11.968
IFN $^{-}$ TNF $^{+}$ CD8	0.066%	10%	11.113
IFN $^{+}$ TNF $^{-}$ CD4	0.035%	35.000%	5.984
IFN $^{+}$ TNF $^{+}$ CD8	0.020%	3.077%	3.419
CCL17^{+} M2	0.014%	2.941%	2.594
Th2	0.013%	0.885%	2.142
PD-L1^{+} M2	0.011%	0.242%	1.848
IFN $^{-}$ TNF $^{+}$ CD4	0.010%	10.000%	1.710
Ki67^{+} CD4	0.010%	10.000%	1.710
IFN $^{+}$ TNF $^{+}$ CD4	0.005%	5.000%	0.855
CD83^{+} DC	0%	0%	0
MHC Class II^{+} DC	0%	0%	0
IL12^{+} CCL17^{-} DC	0%	0%	0
IL12^{-} CCL17^{+} DC	0%	0%	0
IL12^{+} CCL17^{+} DC	0%	0%	0
MHC Class II^{+} M1	0%	0%	0
Th17	0%	0%	0
MHC Class I^{+} M1	0%	0%	0
MHC Class I^{+} DC	0%	0%	0
PD-L1^{+} DC	0%	0%	0
CD83^{+} M1	0%	0%	0
Ki67^{+} Eomes^{+} CD4	0%	0%	0
Ki67^{+} PD-1^{+} CD4	0%	0%	0

Ki67^{+} Eomes^{+} CD8	0%	0%	0
Ki67^{+} PD-1^{+} CD8	0%	0%	0
PD-L1^{+} M1	0%	0%	0

Supplementary Table S11 Bubble Plot Data for Cluster 1 (NT)

Cell Type	Percentage Out of Total Cell	Percentage Out of Subcategory	Cell Density
MHC Class I^{+} M2	6.172%	94.629%	892.674
CD83^{+} M2	2.529%	32.951%	363.034
Th2	2.386%	15.051%	278.477
MHC Class I^{+} M1	2.175%	83.076%	279.648
Th17	2.174%	14.370%	250.664
MHC Class II^{+} M2	2.017%	31.575%	275.280
CD83^{+} DC	2.009%	42.051%	246.398
MHC Class I^{+} DC	1.953%	89.606%	244.027
MHC Class II^{+} DC	1.909%	41.979%	233.243
IFN $^{+}$ TNF $^{+}$ CD4	1.866%	23.807%	221.011
CD83^{+} M1	1.559%	34.609%	192.445
IFN $^{+}$ TNF $^{-}$ CD4	1.390%	25.936%	165.830
MHC Class II^{+} M1	1.133%	23.326%	138.207
IFN $^{-}$ TNF $^{+}$ CD4	0.943%	12.647%	111.797
IFN $^{+}$ TNF $^{+}$ CD8	0.915%	13.255%	108.967
IFN $^{+}$ TNF $^{-}$ CD8	0.718%	17.102%	88.481
Treg	0.665%	11.717%	99.605
IFN $^{-}$ TNF $^{+}$ CD8	0.538%	7.935%	64.446
Ki67^{+} CD8	0.470%	12.827%	57.983
Ki67^{+} CD4	0.457%	9.001%	54.641
Th1	0.376%	7.149%	62.179
PD-L1^{+} M1	0.332%	13.500%	27.823
PD-L1^{+} M2	0.332%	3.495%	53.205
IL12^{+} CCL17^{+} DC	0.251%	26.400%	23.847
Ki67^{+} PD-1^{+} CD4	0.135%	2.738%	16.198
PD-L1^{+} DC	0.123%	25.307%	20.032
CCL17^{+} M2	0.107%	6.049%	11.906
Ki67^{+} PD-1^{+} CD8	0.081%	1.192%	9.503
IL12^{+} M1	0.066%	1.443%	7.646

Ki67^{+} Eomes^{+} CD8	0.040%	1.414%	5.036
Ki67^{+} Eomes^{+} CD4	0.023%	0.318%	2.693
IL12^{+} CCL17^{-} DC	0.010%	0.939%	0.975
IL12^{-} CCL17^{+} DC	0%	0%	0

Supplementary Table S12 Bubble Plot Data for Cluster 2 (M)

Cell Type	Percentage Out of Total Cell	Percentage Out of Subcategory	Cell Density
MHC Class I^{+} M2	7.356%	90.091%	895.840
MHC Class I^{+} M1	4.640%	86.123%	574.462
MHC Class I^{+} DC	3.808%	91.837%	506.950
PD-L1^{+} DC	3.056%	68.798%	385.900
CD83^{+} M1	2.981%	34.861%	341.862
CD83^{+} M2	2.483%	23.334%	291.328
CD83^{+} DC	1.674%	44.601%	198.748
MHC Class II^{+} DC	1.628%	26.807%	183.623
Th2	1.537%	71.000%	196.246
MHC Class II^{+} M1	1.528%	15.192%	171.629
MHC Class II^{+} M2	1.342%	8.001%	151.117
PD-L1^{+} M1	1.142%	45.758%	179.862
PD-L1^{+} M2	1.142%	30.453%	162.932
Treg	0.725%	41.885%	103.867
Ki67^{+} CD8	0.553%	11.991%	81.571
IL12^{+} M1	0.238%	2.474%	28.724
CCL17^{+} M2	0.066%	3.689%	8.464
Th1	0.063%	4.907%	10.768
Th17	0.039%	1.520%	4.619
IFN $^{+}$ TNF $^{-}$ CD8	0.031%	0.865%	4.616
IFN $^{+}$ TNF $^{-}$ CD4	0.012%	6.481%	1.796
IL12^{+} CCL17^{+} DC	0.011%	2.203%	1.282
IFN $^{-}$ TNF $^{+}$ CD8	0.007%	33.406%	1.031
IFN $^{+}$ TNF $^{+}$ CD8	0.003%	0.110%	0.512
Ki67^{+} CD4	0.002%	8.333%	0.261
IL12^{+} CCL17^{-} DC	0%	0%	0
IL12^{-} CCL17^{+} DC	0%	0%	0
IFN $^{-}$ TNF $^{+}$ CD4	0%	0%	0
IFN $^{+}$ TNF $^{+}$ CD4	0%	0%	0

Ki67^{+} Eomes^{+} CD4	0%	0%	0
Ki67^{+} PD-1^{+} CD4	0%	0%	0
Ki67^{+} Eomes^{+} CD8	0%	0%	0
Ki67^{+} PD-1^{+} CD8	0%	0%	0

Supplementary Table S13 Bubble Plot Data for Cluster 3 (G)

**Cell Type**	**Percentage Out of Total Cell**	**Percentage Out of Subcategory**	**Cell Density**
MHC Class I^{+} DC	10.750%	98.860%	1004.918
MHC Class I^{+} M2	9.523%	96.458%	1047.144
IFN $^{+}$ TNF $^{-}$ CD8	5.129%	32.782%	613.402
MHC Class II^{+} DC	4.299%	50.513%	347.225
CD83^{+} DC	3.258%	40.623%	314.512
IFN $^{+}$ TNF $^{-}$ CD4	2.762%	49.013%	347.595
IL12^{+} M1	2.753%	10.768%	236.364
IFN $^{+}$ TNF $^{+}$ CD8	2.688%	19.505%	278.204
CD83^{+} M2	2.659%	26.069%	293.845
MHC Class II^{+} M2	2.656%	27.855%	284.425
MHC Class I^{+} M1	1.976%	26.626%	197.119
MHC Class II^{+} M1	1.750%	15.529%	125.945
CD83^{+} M1	1.630%	22.893%	129.174
IFN $^{+}$ TNF $^{+}$ CD4	1.539%	27.014%	157.156
Ki67^{+} CD8	0.787%	7.269%	96.361
Ki67^{+} CD4	0.701%	11.338%	98.566
Th2	0.612%	25.406%	79.476
IL12^{+} CCL17^{+} DC	0.603%	22.445%	57.091
IFN $^{-}$ TNF $^{+}$ CD8	0.600%	6.331%	81.477
Treg	0.593%	23.300%	78.581
PD-L1^{+} DC	0.584%	6.576%	66.698
Th17	0.468%	18.611%	57.281
PD-L1^{+} M1	0.440%	11.498%	93.236
PD-L1^{+} M2	0.440%	3.669%	51.162
CCL17^{+} M2	0.430%	7.018%	37.937
IFN $^{-}$ TNF $^{+}$ CD4	0.147%	1.827%	20.219
Th1	0.107%	2.623%	15.389
IL12^{-} CCL17^{+} DC	0.079%	6.540%	5.534
Ki67^{+} Eomes^{+} CD8	0.067%	0.462%	9.464

Ki67$^+$ PD-1$^+$ CD4	0.038%	0.606%	5.429
IL12$^+$ CCL17$^-$ DC	0.028%	4.305%	3.060
Ki67$^+$ Eomes$^+$ CD4	0.024%	0.412%	3.493
Ki67$^+$ PD-1$^+$ CD8	0.004%	0.057%	0.595

Supplementary Table S14 Mean Range Table for Cluster 1 (NT), Cluster 2(M), Cluster 3 (G)

**Staining Panel**	**Cell Type**	**Cell Density(cells/mm^{2})**		
		Cluster 1 (NT) (n=3)	Cluster 2 (M) (n=3)	Cluster 3 (G) (n=4)
Oncogenic Panel	PD-1^{+}- PD-1 positive cells	638.1 (212.8-1349.0)	99.6 (67.3-146.0)	137.7 (12.4-300.8)
	PD-L1^{+}- PD-L1 positive cells	2376.6 (0-4215.6)	5859.4 (4083.6-7815.3)	2055.1 (110.4-3491.1)
	IFN $^{+}$- IFN releasing cells	1041.7 (768.8-1487.1)	318.4 (32.9-832.6)	3277.1 (2654.9-4588.5)
	PD-L1^{+} Pan-CK^{+} - Immune suppressive cancer cells	350.6 (0-717.3)	3071.2 (614.7-5834.7)	104.4 (0-217.2)
	LMP1^{+} Pan-CK^{+} - EBV infected cancer cells	91.0 (5.0-153.1)	1172.5 (382.6-2530.4)	83.8 (0-303.5)
	LMP1^{+} PD-L1^{+} Pan-CK^{+} -Immune suppressive EBV infected cancer cells	63.3 (0-96.8)	968.5 (514.9-2041.2)	57.4 (0-200.3)
Myeloid Panel	DEC205^{+}- Dendritic cells	305.2 (12.2-825.4)	542.3 (80.8-856.7)	1007.5 (106.7-2219.7)
	MHC Class I^{+} DEC205^{+} -Antigen presenting dendritic cells	244.0 (11.1-643.1)	507.0 (72.3-596.5)	1004.9 (103.9-2219.7)
	MHC Class II^{+} DEC205^{+} -Antigen presenting dendritic cells	233.2 (3.3-684.1)	183.6 (0.8-538.4)	347.2 (86.1-720.7)
	PD-L1^{+} DEC205^{+} - Immune suppressive dendritic cells	20.0 (0-59.0)	385.9 (46.9-657.9)	66.7 (0-177.1)
	CD83^{+} DEC205^{+} - Activated dendritic cells	246.4 (1.1-714.3)	198.7 (45.4-469.8)	314.5 (83.1-747.1)
	CD68^{+} CD163^{-} - M1 cells	345.3 (187.8-633.4)	637.1 (364.6-1142.2)	873.7 (432.4-1522.4)
	MHC Class I^{+} CD68^{+} CD163^{-} - Antigen presenting M1 cells	279.6 (140.1-489.9)	574.5 (272.1-1108.3)	197.1 (107.6-329.6)
	MHC Class II^{+} CD68^{+} CD163^{-} - Antigen presenting M1 cells	138.2 (6.7-401.4)	171.6 (0-511.8)	125.9 (35.4-293.4)
	PD-L1^{+} CD68^{+} CD163^{-} -Immune suppressive M1 cells	27.8 (0-59.0)	179.9 (22.6-276.9)	93.2 (1.9-187.9)
	CD83^{+} CD68^{+} CD163^{-} -Activated M1 cells	192.4 (5.6-537.4)	341.9 (39.2-939.6)	129.2 (26.2-312.6)
	CD163^{+}- M2 cells	977.8 (242.3-1944.3)	965.0 (254.6-1984.9)	1074.3 (478.3-2241.3)
	MHC Class I^{+} CD163^{+}- Antigen presenting M2 cells	892.7 (239.0-1713.9)	895.8 (214.6-1871.9)	1047.1 (444.6-2239.8)
	MHC Class II^{+} CD163^{+} -Antigen presenting M2 cells	275.3 (50.0-414.3)	151.1 (1.5-445.6)	284.4 (72.1-511.4)
	PD-L1^{+} CD163^{+} - Suppressive M2 cells	53.2 (5.6-150.8)	162.9 (88.8-292.3)	51.2 (0-144.8)
	CD83^{+} CD163^{+} - Activated M2 cells	363.0 (24.5-652.5)	291.3 (45.4-726.5)	293.8 (101.1-668.5)
	CD4^{+}-T helper cells	965.0	136.2	569.4

Lymphoid and Functional Panel		(415.0-1987.8)	(109.2-161.3)	(52.4-1289.9)
	T-bet^{+} CD4^{+}-Th1	62.2 (4.2-122.8)	10.8 (0.77-30.0)	15.4 (0-40.4)
	GATA3^{+} CD4^{+}-Th2	278.5 (19.6-785.9)	196.2 (53.1-309.6)	79.4 (7.0-168.2)
	ROR T^{+} CD4^{+}-Th17	250.7 (0-710.5)	4.6 (0-11.6)	57.3 (12.1-146.0)
	FOXP3^{+} CD4^{+}-Treg	99.6 (24.9-152.2)	103.9 (74.6-140.7)	78.6 (7.0-244.1)
	Ki67^{+} CD4^{+} -Proliferating T helper cells	54.6 (17.5-93.2)	0.3 (0-0.8)	98.6 (0-281.0)
	Ki67^{+} PD-1^{+} CD4^{+} -Proliferating suppressive T helper cells	16.2 (5.6-28.3)	0 (0-0)	5.4 (0-16.2)
	Ki67^{+} Eomes^{+} CD4^{+} - Proliferating Eomes expressing T helper cells	2.7 (0-7.5)	0 (0-0)	3.5 (0-10.0)
	IFN $^{+}$ TNF CD4^{+}- IFN releasing T helper cells	165.8 (51.3-319.7)	1.8 (0-5.4)	347.6 (51.8-1045.4)
	IFN $^{-}$ TNF CD4^{+}- TNF T releasing helper cells	111.8 (11.9-224.3)	0 (0-0)	20.2 (0-64.7)
	IFN $^{+}$TNF CD4^{+}- IFN and TNF releasing T helper cells	221.0 (10.0-386.8)	0 (0-0)	157.2 (2.9-446.7)
	CD8^{+}- CD8 positive T cells	1453.3 (1299.3-1604.6)	982.0 (360.7-1471.7)	1644.1 (909.1-2575.3)
	Ki67^{+} CD8^{+}- Proliferating CD8 positive T cells	58.0 (37.9-68.2)	81.6 (0-137.0)	96.4 (27.4-191.8)
	Ki67^{+} PD-1^{+} CD8^{+} - Proliferating suppressive CD8 positive T cells	9.5 (0-23.6)	0 (0-0)	0.6 (0-2.4)
	Ki67^{+} Eomes^{+} CD8^{+} - Proliferating CD8 positive memory T cells	5.0 (2.0-9.4)	0 (0-0)	9.5 (0-33.1)
	IFN $^{+}$ TNF CD8^{+}- IFN releasing CD8 positive T cells (non-TNF releasing)	88.5 (25.3-154.4)	4.6 (0-10.8)	613.4 (98.7-1647.4)
	IFN $^{-}$ TNF CD8^{+}- TNF releasing CD8 positive T cells (non- IFN releasing)	64.4 (11.3-141.1)	1.0 (0-2.3)	81.5 (5.9-202.1)
	IFN $^{+}$TNF CD8^{+}- IFN and TNF CD8 positive T cells	109.0 (10.6-184.3)	0.5 (0-1.5)	278.2 (15.6-721.3)
	IFN $^{+}$ TNF - IFN releasing cells (non-TNF releasing)	935.2 (442.7-1569.6)	378.6 (79.3-822.6)	2820.1 (852.3-5989.8)
	IFN $^{-}$ TNF - TNF releasing cells (non- IFN releasing)	663.6 (294.7-904.8)	227.6 (104.7-448.9)	1059.8 (58.0-1694.3)
	IFN $^{+}$TNF - IFN and TNF releasing cells	457.8 (105.7-728.7)	82.4 (30.8-142.6)	1315.2 (734.0-2371.9)
	IFN $^{-}$TNF - Non-IFN and non-TNF releasing cells	10255.4 (96252.3-11265.2)	14404.5 (14338.6-14484.0)	5636.2 (2725.9-10725.0)
	PD-1^{+}- PD-1 positive cells	350.8 (292.1-401.7)	16.2 (9.2-27.7)	76.4 (2.0-220.4)
Refinement Molecular Panel	IL12^{+} - IL12 releasing cells	203.2 (68.2-389.4)	308.3 (33.0-513.3)	1032.0 (295.8-2130.2)
	IL12^{+} CD68^{+} CD163^{-} - IL12 releasing M1 cells	7.6 (1.5-9.7)	28.7 (6.9-52.3)	236.4 (13.5-621.6)

	IL12^{+} CCL17^{-} DEC205^{+} - IL12 releasing dendritic cells	23.8 (1.9-67.2)	1.3 (0-2.3)	57.1 (3.9-173.1)
	CCL17^{+} - CCL17 releasing cells	355.2 (114.0-739.0)	279.8 (27.7-437.1)	461.8 (18.0-855.5)
	CCL17^{+} CD163^{+} - CCL17 releasing M2 cells	11.9 (8.8-15.2)	8.5 (3.1-15.4)	37.9 (0.9-77.7)
	IL12^{-} CCL17^{+} DEC205^{+} -CCL17 releasing dendritic cells	1.0 (0-2.9)	0 (0-0)	3.1 (0-4.6)
	IL12^{+} CCL17^{+} DEC205^{+} - IL12 and CCL17 releasing dendritic cells	0 (0-0)	0 (0-0)	5.5 (0-9.0)
	CD137^{+}- Immune activation marker	134.4 (6.8-212.3)	44.9 (0-81.6)	390.7 (199.2-800.3)
	CD137^{+} CD68^{+} CD163^{-} - CD137 positive M1 cells	15.7 (0-45.7)	1.5 (0-3.8)	31.0 (4.7-87.4)
	CD137^{+} IL12^{+} CD68^{+} CD163^{-} - CD137 positive IL12 releasing M1 cells	0 (0-0)	0.3 (0-0.8)	6.6 (0.9-14.0)
	CD137^{+} IL12^{-} CD68^{+} CD163^{-} - CD137 positive non-IL12 releasing M1 cells	15.7 (0-45.7)	1.2 (0-3.1)	24.4 (2.3-73.4)
	CD137^{+} CD163^{+} - CD137 positive M2 cells	50.3 (2.9-143.3)	20.0 (0-60.0)	107.2 (18.0-223.4)
	CD137^{+} CCL17^{+} CD163^{+} -CD137 positive CCL17 releasing M2 cells	2.2 (1.0-4.4)	0 (0-0)	7.9 (0.9-21.1)
	CD137^{+} CCL17^{-} CD163^{+} -CD137 positive non-CCL17 releasing M2 cells	48.1 (1.9-138.9)	20.0 (0-60.0)	99.3 (13.3-215.2)
	CD137^{+} CD8^{+}- CD137 positive CD8 positive T cells	15.9 (2.9-36.5)	6.7 (0-18.5)	78.5 (43.0-98.9)
	CD137^{+} IFN $^{+}$ CD8^{+} - CD137 positive IFN releasing CD8 positive T cells	10.5 (2.3-26.3)	0 (0-0)	20.0 (1.2-59.4)
	CD137^{+} IFN $^{-}$ CD8^{+} - CD137 positive non-IFN releasing CD8 positive T cells	5.4 (0-10.2)	6.7 (0-18.5)	58.5 (19.8-97.7)
	CD137^{+} DEC205^{+} - CD137 positive dendritic cells	15.6 (1.0-42.4)	0 (0-0)	16.7 (4.7-43.0)

Supplementary Table S15 Mean Range Table for Outlier 1 (NT-P) and Outlier 2 (NT-I)

Staining Panel	Cell Type	Cell Density(cells/mm^2)	
		Outlier 2 (NT-I) (n=1)	Outlier 1 (NT-P) (n=1)
Oncogenic Panel	PD-1$^+$- PD-1 positive cells	202.9	61.8
	PD-L1$^+$- PD-L1 positive cells	789.6	5.7
	IFN $^+$- IFN releasing cells	918.0	2718.7
	PD-L1$^+$ Pan-CK$^+$ - Immune suppressive cancer cells	207.6	0
	LMP1$^+$ Pan-CK$^+$ - EBV infected cancer cells	169.2	365.2
	LMP1$^+$ PD-L1$^+$ Pan-CK$^+$ -Immune suppressive EBV infected cancer cells	158.2	0
Myeloid Panel	DEC205$^+$- Dendritic cells	0	376.7
	MHC Class I$^+$ DEC205$^+$ -Antigen presenting dendritic cells	0	266.3
	MHC Class II$^+$ DEC205$^+$ -Antigen presenting dendritic cells	0	246.6
	PD-L1$^+$ DEC205$^+$ - Immune suppressive dendritic cells	0	0
	CD83$^+$ DEC205$^+$ -Activated dendritic cells	0	53.9
	CD68$^+$ CD163$^-$ -M1 cells	0	133.6
	MHC Class I$^+$ CD68$^+$ CD163$^-$ - Antigen presenting M1 cells	0	112.2
	MHC Class II$^+$ CD68$^+$ CD163$^-$ - Antigen presenting M1 cells	0	35.1
	PD-L1$^+$ CD68$^+$ CD163$^-$ -Immune suppressive M1 cells	0	0
	CD83$^+$ CD68$^+$ CD163$^-$ -Activated M1 cells	0	34.2
	CD163$^+$- M2 cells	762.3	774.0
	MHC Class I$^+$ CD163$^+$-Antigen presenting M2 cells	754.9	651.6
	MHC Class II$^+$ CD163$^+$ -Antigen presenting M2 cells	23.1	200.4
	PD-L1$^+$ CD163$^+$ -Suppressive M2 cells	1.8	0
	CD83$^+$ CD163$^+$ - Activated M2 cells	102.6	36.8
Lymphoid and Functional Panel	CD4$^+$- T helper cells	129.5	2654.3
	T-bet$^+$ CD4$^+$-Th1	15.0	12.5
	GATA3$^+$ CD4$^+$-Th2	2.1	591.2
	ROR T$^+$ CD4$^+$-Th17	0	110.8
	FOXP3$^+$ CD4$^+$-Treg	22.5	105.3
	Ki67$^+$ CD4$^+$ - Proliferating T helper cells	1.7	70.3
	Ki67$^+$ PD-1$^+$ CD4$^+$ - Proliferating suppressive T helper cells	0	0

	Ki67$^+$ Eomes$^+$ CD4$^+$ - Proliferating Eomes expressing T helper cells	0	1.7
	IFN $^+$ TNF CD4$^+$- IFN releasing T helper cells	6.0	1497.4
	IFN $^-$ TNF CD4$^+$- TNF T releasing helper cells	1.7	96.7
	IFN $^+$ TNF CD4$^+$- IFN and TNF releasing T helper cells	0.9	234.8
	CD8$^+$- CD8 positive T cells	528.9	5908.3
	Ki67$^+$ CD8$^+$ - Proliferating CD8 positive T cells	65.8	56.2
	Ki67$^+$ PD-1$^+$ CD8$^+$ - Proliferating suppressive CD8 positive T cells	0	0
	Ki67$^+$ Eomes$^+$ CD8$^+$ - Proliferating CD8 positive memory T cells	0.9	3.3
	IFN $^+$ TNF CD8$^+$- IFN releasing CD8 positive T cells (non-TNF releasing)	12.0	1224.6
	IFN $^-$ TNF CD8$^+$- TNF releasing CD8 positive T cells (non- IFN releasing)	11.1	38.0
	IFN $^+$ TNF CD8$^+$- IFN and TNF CD8 positive T cells	3.4	126.5
	IFN $^+$ TNF - IFN releasing cells (non-TNF releasing)	590.7	4129.3
	IFN $^-$ TNF - TNF releasing cells (non- IFN releasing)	417.2	913.7
	IFN $^+$ TNF - IFN and TNF releasing cells	23.9	504.4
	IFN $^-$ TNF - Non-IFN and non-TNF releasing cells	15871.8	11012.9
	PD-1$^+$- Immune suppressive cells	62.4	41.3
Refinement Molecular Panel	IL12$^+$ -IL12 releasing cells	439.6	4194.7
	IL12$^+$ CD68$^+$ CD163$^-$ - IL12 releasing M1 cells	29.8	90.7
	IL12$^+$ CCL17$^-$ DEC205$^+$ - IL12 releasing dendritic cells	0	349.6
	CCL17$^+$ - CCL17 releasing cells	373.5	143.7
	CCL17$^+$ CD163$^+$ - CCL17 releasing M2 cells	2.6	5.1
	IL12$^-$ CCL17$^+$ DEC205$^+$ -CCL17 releasing dendritic cells	0	0
	IL12$^+$ CCL17$^+$ DEC205$^+$ - IL12 and CCL17 releasing dendritic cells	0	5.1
	CD137$^+$- Immune activation marker	199.7	18.3
	CD137$^+$ CD68$^+$ CD163$^-$ - CD137 positive M1 cells	9.1	3.1
	CD137$^+$ IL12$^+$ CD68$^+$CD163$^-$ -CD137 positive IL12 releasing M1 cells	0	0
	CD137$^+$ IL12$^-$ CD68$^+$CD163$^-$ -CD137 positive non-IL12 releasing M1 cells	9.1	3.1

	$CD137^{+}$ $CD163^{+}$ - CD137 positive M2 cells	0	7.1
	$CD137^{+}$ $CCL17^{+}$ $CD163^{+}$ -CD137 positive CCL17 releasing M2 cells	0	0
	$CD137^{+}$ $CCL17^{-}$ $CD163^{+}$ -CD137 positive non-CCL17 releasing M2 cells	0	7.1
	$CD137^{+}$ $CD8^{+}$- CD137 positive CD8 positive T cells	6.5	3.1
	$CD137^{+}$ IFN^{+} $CD8^{+}$ - CD137 positive IFN releasing CD8 positive T cells	0	1.0
	$CD137^{+}$ IFN^{-} $CD8^{-}$ - CD137 positive non-IFN releasing CD8 positive T cells	6.5	2.1
	$CD137^{+}$ $DEC205^{+}$ - CD137 positive dendritic cells	0	2.0

Supplementary Table S16 R Package

R analysis	Library
Bar Chart, Box Plot, Dot Plot (Monomarker)	ggplot2, ggpubr, dplyr, rstatix
Bubble Plot, Line Dot Plot, Dot Plot (Cell Type), Histogram	ggplot2
Distance analysis	stingr, phenoptr, dplyr
Eclipse Correlation	ggcorrplot2, plyr, rstatix, ggplot2
Genetic Analysis	GEOquery, limma, tidyverse, data.table
Radar Chart	Fmsb
Rose Plot	ggpubr, ggplot2
Scatter Pie Chart	ggplot2, scatterpie
t-SNE	Rtsne, ggplot2, caret
Network Analysis	network, sna, ggplot 2, GGally
Circus Plot	Tidyverse, circlize, graphics
